# Structural Insights into the Assembly of the African Swine Fever Virus Inner Capsid

**DOI:** 10.1128/jvi.00268-23

**Published:** 2023-05-16

**Authors:** Haining Li, Qi Liu, Luyuan Shao, Ye Xiang

**Affiliations:** a Center for Infectious Disease Research, Beijing Frontier Research Center for Biological Structure & Beijing Advanced Innovation Center for Structural Biology, Department of Basic Medical Sciences, School of Medicine, Tsinghua University, Beijing, China; Northwestern University Feinberg School of Medicine

**Keywords:** African swine fever virus, nucleocytoplasmic large DNA viruses, cryogenic electron microscopy, inner capsid assembly, new viral capsid scaffold, virion structure

## Abstract

African swine fever virus (ASFV), the cause of a highly contagious hemorrhagic and fatal disease of domestic pigs, has a complex multilayer structure. The inner capsid of ASFV located underneath the inner membrane enwraps the genome-containing nucleoid and is likely the assembly of proteolytic products from the virally encoded polyproteins pp220 and pp62. Here, we report the crystal structure of ASFV p150_△NC_, a major middle fragment of the pp220 proteolytic product p150. The structure of ASFV p150_△NC_ contains mainly helices and has a triangular plate-like shape. The triangular plate is approximately 38 Å in thickness, and the edge of the triangular plate is approximately 90 Å long. The structure of ASFV p150_△NC_ is not homologous to any of the known viral capsid proteins. Further analysis of the cryo-electron microscopy maps of the ASFV and the homologous faustovirus inner capsids revealed that p150 or the p150-like protein of faustovirus assembles to form screwed propeller-shaped hexametric and pentametric capsomeres of the icosahedral inner capsids. Complexes of the C terminus of p150 and other proteolytic products of pp220 likely mediate interactions between the capsomeres. Together, these findings provide new insights into the assembling of ASFV inner capsid and provide a reference for understanding the assembly of the inner capsids of nucleocytoplasmic large DNA viruses (NCLDV).

**IMPORTANCE** African swine fever virus has caused catastrophic destruction to the pork industry worldwide since it was first discovered in Kenya in 1921. The architecture of ASFV is complicated, with two protein shells and two membrane envelopes. Currently, mechanisms involved in the assembly of the ASFV inner core shell are less understood. The structural studies of the ASFV inner capsid protein p150 performed in this research enable the building of a partial model of the icosahedral ASFV inner capsid, which provides a structural basis for understanding the structure and assembly of this complex virion. Furthermore, the structure of ASFV p150_△NC_ represents a new type of fold for viral capsid assembly, which could be a common fold for the inner capsid assembly of nucleocytoplasmic large DNA viruses (NCLDV) and would facilitate the development of vaccine and antivirus drugs against these complex viruses.

## INTRODUCTION

**FIG 1:**
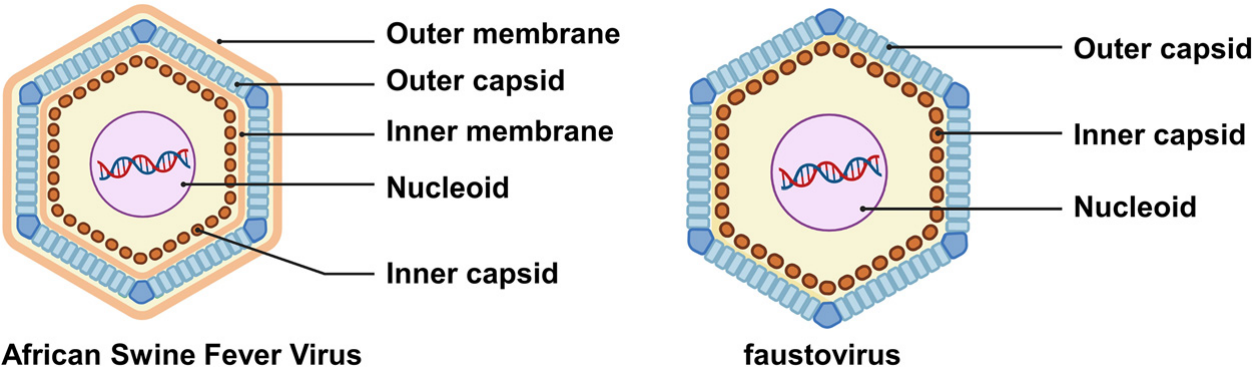
Schematic diagrams showing the complex multilayer architectures of ASFV and faustovirus.

African swine fever virus (ASFV), the cause of lethal infectious diseases in domestic pigs and wild boars, is a great threat to the pork industry (1). There are still no effective vaccines or drugs for the control and treatment of ASFV infection. African swine fever virus is a giant icosahedral virus with a diameter of ~2,000 Å and has a complex multiple-layer structure encapsidating a double-stranded DNA (dsDNA) genome that encodes more than 150 proteins (2). From the outside to the inside, the virion of ASFV has an outer membrane, a capsid of viral protein p72, an inner membrane, an inner capsid that is approximately 50 Å thick, and a nucleoid of the dsDNA genome and viral protein complex (Fig. 1) (3–7). The icosahedral outer capsid (T = 277, h = 7, k = 12) of ASFV is composed of 2,760 pseudohexametric capsomeres and 12 pentametric capsomeres that form the trisymmetrons and pentasymmetrons. The 5-fold vertices of the capsid are occupied by 12 pentametric capsomeres (3–5). Each pseudohexametric capsomere contains three molecules of the major capsid protein p72 (6). The penton protein H240R forms the pentametric capsomeres (4). The capsid contains also three minor proteins, likely including the zipper protein M1249L that links adjacent pentasymmetrons, p17 that anchors the p72 capsomeres, and p49 that anchors the H240 capsomeres (3, 4). The structure of p72 determined by cryo-electron microscopy (cryo-EM) showed a classic double jelly roll fold (6), a common fold found in many icosahedral viral capsid proteins (3, 4, 8). ASFV is the only known member of the family *Asfaviridae* (9). The structure and genome sequence of ASFV are similar to these of the faustovirus (Fig. 1), an unclassified giant dsDNA virus (10).

**FIG 2:**
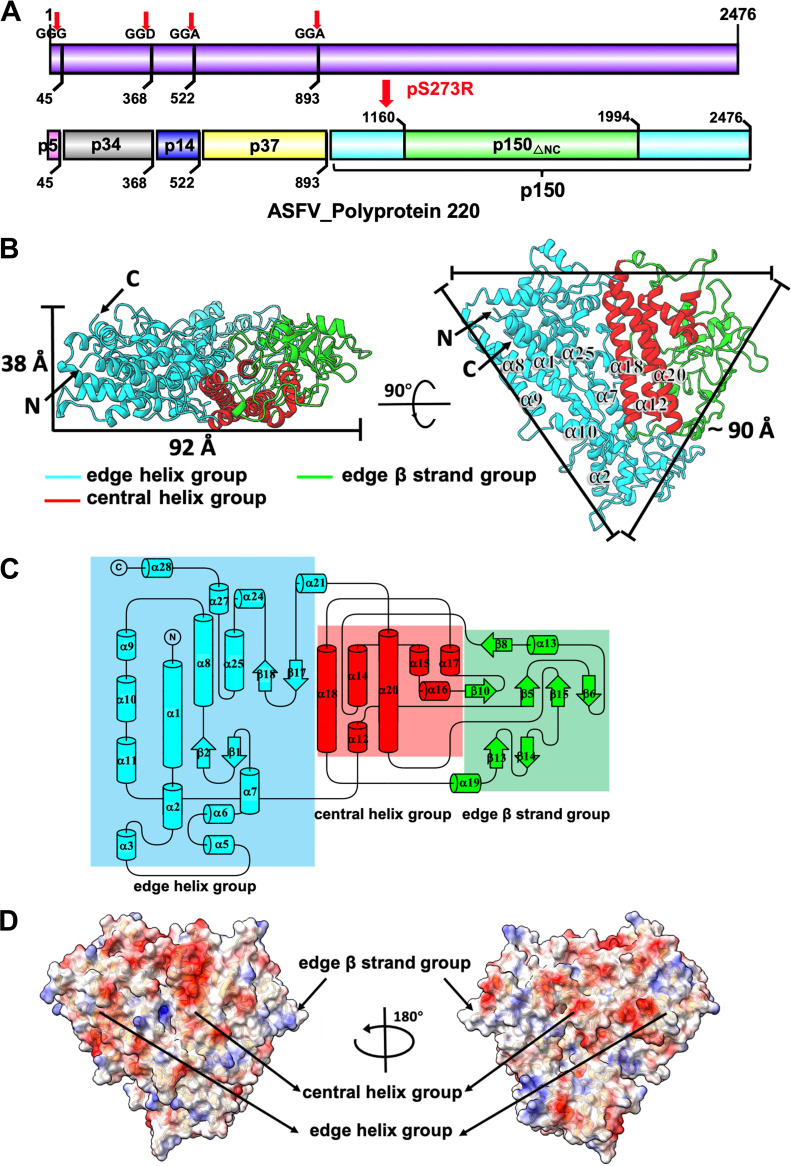
Overall structure of p150_△NC_. (A) Schematic diagrams (41) showing the organization and proteolytic process of the polyprotein pp220 by the viral-encoded protease pS273R. The precursor, pp220, is colored purple. The proteolytic products p5, p34, p14, p37, p150, and p150_△NC_ are colored pink, gray, blue, yellow, cyan, and green, respectively. The cleavage sites are shown above the diagram and indicated by red arrows. (B) Overall structure of p150_△NC_. (B, Right) Front view. (B, Left) Top view. The structure is shown as a cartoon with the N terminus, C terminus, and some main helices labeled. The helices in the central helix group are colored red, the helices in the edge helix group are colored cyan, and the β strands in the edge β strand group are colored green. (C) Diagrams (42) showing the topology of p150_△NC_. The main helices and strands of p150_△NC_ are shown in the topology. The helices in the central helix group are colored red, the helices in the edge helix group are cyan, and the β strands in the edge β strand group are green. (D) Surface electrostatic potential of p150_△NC_. The surface is colored according to the surface electrostatic potential, with red for negative potential and blue for positive potential.

The 50-Å-thick inner capsid of ASFV is part of the inner core shell and has a diameter of 180 nm (3, 4). The inner core shell has interactions with both the inner envelope and the nucleoid. Repression of the viral polyprotein pp220 production by using an Escherichia coli lac operator-repressor system leads to the production of unusual ASFV particles that do not have the inner core shell and the nucleoid (11). Similar repression of the viral polyproteins pp62 leads to the production of ASFV particles that do not have the intact nucleoid (12). The two polyproteins, pp220 and pp62, are proteolytically processed during virus maturation by a viral-encoded protease, pS273R, that recognizes a sequence pattern of Gly-Gly-X (X is often an amino acid residue with a hydrophobic side chain) and cleaves the polypeptide chain after the second Gly residue (13, 14). Proteolytic cleavage of pp220 produces p5, p34, p14, p37, and p150 (Fig. 2A), while proteolytic cleavage of pp62 produces p15, p35 and p8. Among these, p150, which contains residues 894 to 2476 of pp220, is the most abundant virion protein (in the percentage of the virion protein mass) after the major outer capsid protein p72 (15–17). Subviral localization of the polyprotein products showed that they are located mainly in the core shell (16). The inner capsid is likely assembled from the proteolytic products of pp220, with p150 as the major inner capsid protein (15, 17).

The inner capsid of ASFV was reconstructed to a resolution of 9 Å and showed a T = 19 (h = 2, k = 3) icosahedral shell with 180 hexametric capsomeres and 12 pentametric capsomeres (4, 5). Similarly, the homologous faustovirus has an inner capsid (Fig. 1) of similar size and encodes polyproteins that are homologous to pp220 and pp62, respectively (10, 18). The detailed mechanisms of the assembly of the inner capsids of these giant viruses are not clearly understood in general.

Here, we present the crystal structure of a major fragment of the ASFV inner capsid protein p150 at a resolution of 2.4 Å. Fitting of the crystal and predicted structures into cryo-EM maps of the inner capsids of ASFV and faustovirus indicates that p150 assembles to form hexametric and pentametric capsomeres and constitutes the major part of the inner capsid. The structure of p150 shows no similarities to any of the currently known viral capsid proteins, thus presenting a new fold for the assembly of the viral capsid.

## RESULTS

### Structure determination.

We produced the full-length p150, which contains residues 894 to 2476 of pp220 in HEK293F cells. The residues of p150 are renumbered from 1 to 1583 in the following descriptions. We found that most of the full-length p150 was in the insoluble part of the cell lysate. A small portion of the recombinant p150 was soluble and can be purified. The purified p150 shows a molecular weight of ~180 kDa on the SDS-PAGE gels (see Fig. S1A in the supplemental material). A gradient fixation (GraFix) (19) analysis with a 10 to 45% (wt/vol) sucrose gradient and 0.15% glutaraldehyde showed that the soluble recombinant p150 is located in most layers of the gradient, indicating that soluble recombinant p150 is most likely in complex oligomeric forms (Fig. S1B). However, the recombinant p150 is not stable, and most of the protein was degraded into a stable fragment with a molecular weight of ~80 kDa after storage at 4°C for 1 week (Fig. S1C). The stable segment of p150 was subjected to mass spectral analysis, and the results showed that all the peptide hits are located in the middle of p150 from residue 226 to residue 1077 (Fig. S2A). According to the mass spectral analysis, we thus made five different truncations that contain residues 216 to 1079, 227 to 1011, 227 to 1101, 267 to 1011, and residues 267 to 1101 of p150, respectively (Fig. S2B). The truncation mutant containing residues 267 to 1101 has a relatively higher expression level (Fig. S2B). We then chose p150_△NC_ (residues 267 to 1101) for structural studies.

The elution volume of p150_△NC_ from size exclusion chromatography (SEC) indicates that p150_△NC_ is a monomer in solution (Fig. S3). We obtained a crystal of p150_△NC_ that diffracted to a resolution of 2.4 Å. The crystal belongs to the space group P1 with cell dimensions a = 54.44 Å, b = 65.60 Å, c = 115.01 Å, α = 99.208°, β = 92.705°, and γ = 90.162° (Table S1). Solvent content analysis of the crystal suggested that two p150_△NC_ molecules are most likely in the asymmetric unit. The phase problem was solved by molecular replacement using a model generated by AlphaFold (20). Approximately 54.7% of the residues in the predicted structure have a confidence score of more than 70 (Fig. S4), although the multiple-sequence alignments from AlphaFold showed that the sequence of p150_△NC_ does not have significant homology to any of the known structures. The initial molecular replacement solution showed a log-likelihood gain (LLG) value of 437.6 and an R factor of 0.466. The initial phases were then improved by iterative direct method phase extension with Oasis (21), density modifications with Parrot (22), and automatic model building with Buccaneer (23). The final atomic model of p150_△NC_ contains residues 268 to 1074. Two disordered loops (503 to 514 and 1075 to 1101) were not built due to poor densities. The model was refined to *R* and *R*_free_ values of 0.2454 and 0.2891, respectively (Table S1). The two molecules in the asymmetric unit have similar structures, with a root mean square deviation (RMSD) of 0.188 Å. In addition, the final model and the initial model from AlphaFold prediction have an RMSD of 0.834 Å, indicating the amazing accuracy of the prediction.

### Structure of p150_△NC_.

The structure of p150_△NC_ has an open-sheet shape resembling an upside-down triangle, of which the thickness is approximately 38 Å and each edge is approximately 90 Å long. The structure of p150_△NC_ is rich in helices and consists of 5 long helices, 23 short helices, 16 3_10_ helices, and a few short β strands (Fig. 2B). The helices are clustered into two major groups, with one group (central helix group) oriented roughly vertical to one edge of the triangle and containing long helices α12, α14, α15 to α18, and α20 (Fig. 2B and C). The other group (edge helix group) is oriented along one edge of the triangle and contains long helices α1, α2, α7 to α11, and α25 (Fig. 2B and C). The short β strands, together with several short loops (edge β strand group), are mainly located near the adjacent edge to the edge helix group (Fig. 2B and C). A DALI search for homologous structures shows that the structure of p150_△NC_ shares no structural similarity to these of other proteins or the known viral capsid proteins, suggesting that the structure of p150_△NC_ is likely a new fold for the assembly of viral capsids. The surface electrostatic potential of p150_△NC_ is analyzed, and the results show that positively charged and negatively charged residues are relatively evenly distributed on the surface of p150_△NC_ (Fig. 2D).

### Assembly of p150_△NC_ in the inner core shell.

**FIG 3:**
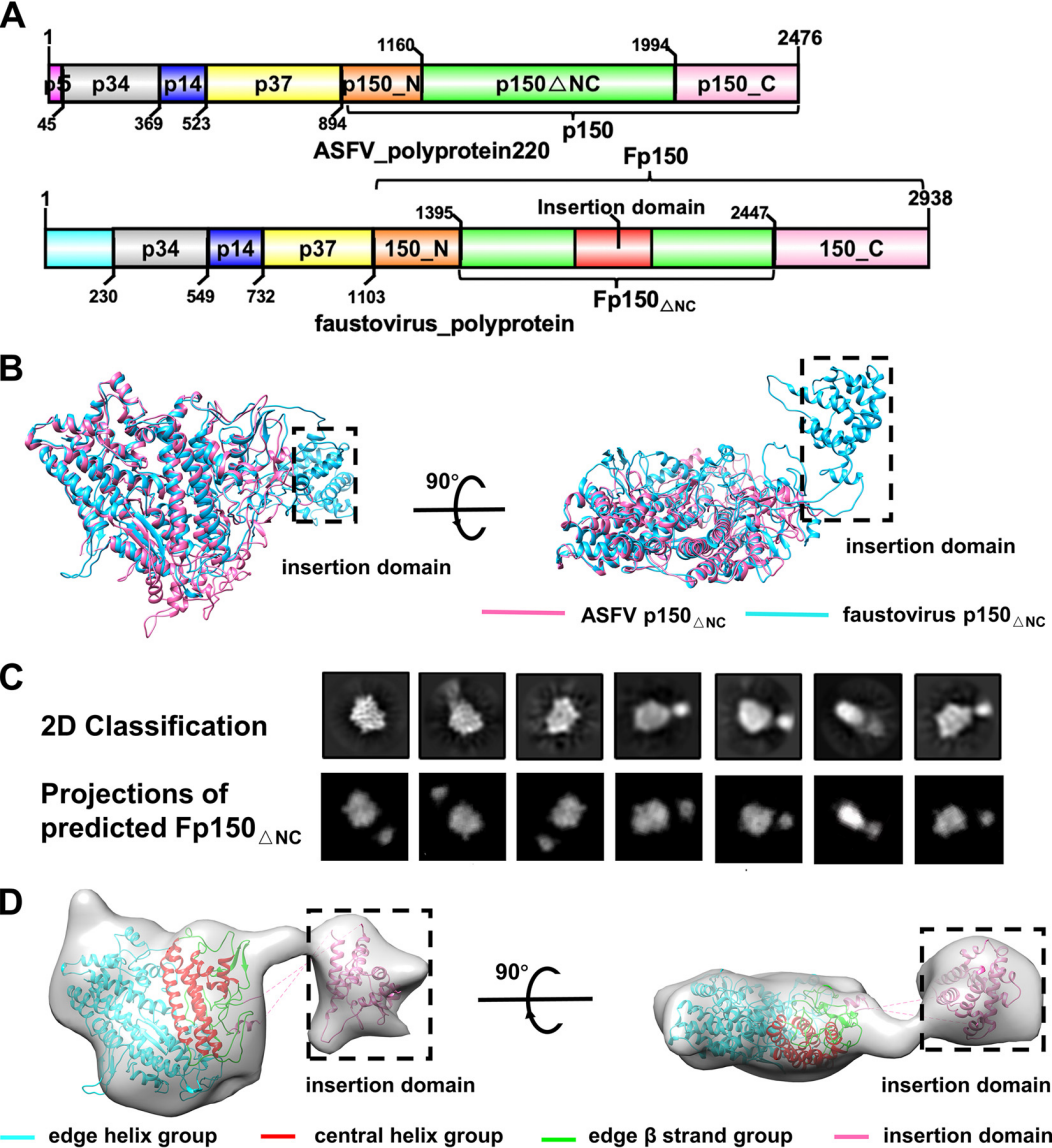
Sequence and structure comparisons of ASFV p150_△NC_ and faustovirus p150_△NC_. (A) Schematic diagrams (41) showing the proteolytic products of the ASFV polyprotein pp220 and the homologous faustovirus polyprotein Fp150_△NC_. (B) Superimpositions of the ASFV p150_△NC_ structure and the predicted Fp150_△NC_ structure. The crystal structure of ASFV p150_△NC_ is colored hot pink. The predicted structure of Fp150_△NC_ is colored deep sky blue. The insertion domain of Fp150_△NC_ is marked with dashed lines. (C) Comparisons between the 2D class averages of Fp150_△NC_ and the projections generated from the predicted Fp150_△NC_ structure. (D) Predicted structure of Fp150_△NC_ fitted in the cryo-EM map of Fp150_△NC_, which was set at a contouring level of 3.5 σ. The central helix group, edge helix group, and edge β strand groups of one monomer are colored red, cyan, and green, respectively. The insertion domain is colored hot pink.

Given that p150 is the major component of the inner capsid, we hypothesized that p150 could constitute the hexametric and pentametric capsomeres. Thus, we tried fitting the crystal structure of p150_△NC_ into the low-resolution cryo-EM map of the ASFV inner capsid (4). However, the quality of the ASFV density map is not good enough for unambiguously locating the p150_△NC_ molecules. The ASFV polyprotein pp220 shows a sequence identity of 27.01% to that of a faustovirus polyprotein (GenPept accession no. SMH63597). The corresponding fragment in the polyprotein of faustovirus (Fp150_△NC_) has an additional insertion domain of 262 amino acids (residues 1782 to 2043 in the polyprotein) in the middle compared to that of p150_△NC_ (Fig. 3A and Fig. S5). Except for the insertion domain, the rest of Fp150_△NC_ has approximately 21% of the residues identical to those of p150_△NC_ (Fig. S5). As expected, structure prediction with AlphaFold showed that the structure of Fp150_△NC_ is highly similar to that of p150_△NC_ (Fig. 3B). The predicted insertion domain of Fp150_△NC_ has a globular structure of 12 helices and protrudes from the β strand group edge of Fp150_△NC_ (Fig. 3B and Fig. S4). We further produced the recombinant protein Fp150_△NC_ (Fig. S6A and B). SEC analysis showed that Fp150_△NC_ is also a monomer in solution (Fig. S6C). Cryo-EM analysis of Fp150_△NC_ showed a structure consistent with the AlphaFold prediction, with differences only in the hinge angle between the insertion domain and the p150_△NC_-like plate (Fig. 3C and D).

**FIG 4:**
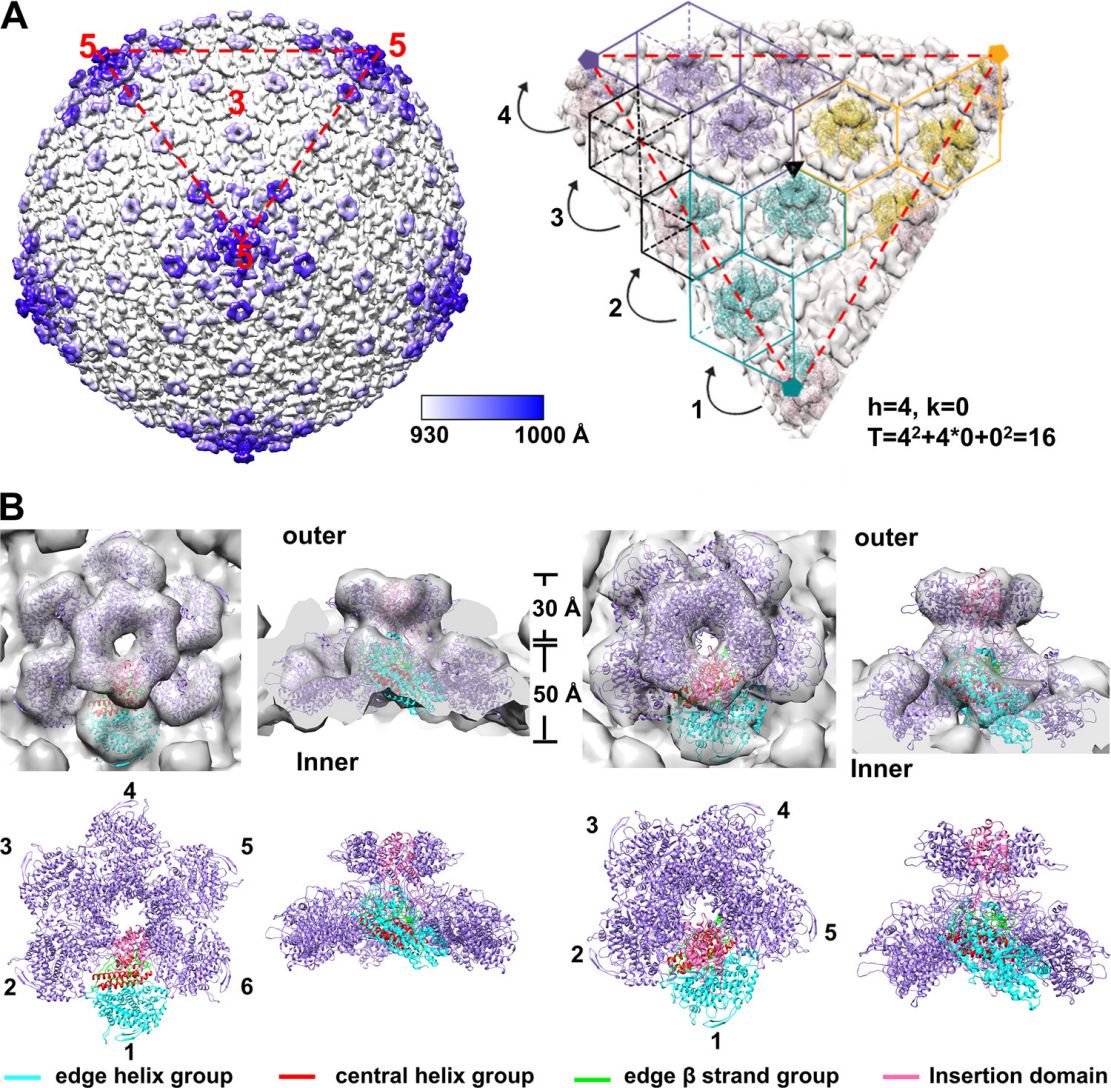
Fitted faustovirus Fp150_△NC_ in the cryo-EM map of the faustovirus inner core shell. (A) Partial inner core shell structure of faustovirus obtained by fitting the hexametric and pentametric capsomeres of Fp150_△NC_ into the density map of the faustovirus inner protein shell (EMD-8145) (10). Voxels of the map are colored according to their radical distances to the particle center. The color scale indicates the color scheme for voxels at different distances to the particle center. One triangular facet is indicated by the red triangle in dashed lines. Positions of the icosahedral symmetr*y* axes are indicated with numbers. The subunits in different asymmetry units are in different colors. The h vectors of the hexametric capsomeres are shown on a stripped triangular facet at the right. (B) The hexametric and pentametric capsomeres constituted by Fp150_△NC_ are fitted into the density map (EMD-8145) of the faustovirus inner protein shell, which was set at a contouring level of 1.9 σ (10). The central helix group, edge helix group, and edge β strand groups of one monomer are colored red, cyan, and green, respectively. The insertion domains are colored hot pink. Other monomers are colored purple.

The cryo-EM map (EMD-8145) of faustovirus showed that the inner capsid is a T = 16 quasi-icosahedron (Fig. 4A), of which the hexametric and pentametric capsomeres are visible and featured with a ring-shaped top structure (10). The location of the monomers in the capsomeres of the faustovirus inner capsid shell is also clearly distinguishable (Fig. 4A and B). The predicted structure of Fp150_△NC_ could be well fitted into the density map of the hexametric and pentametric capsomeres, with the protruding insertion domains forming the top rings (Fig. 4A). The correlation coefficient (CC) between the fitted hexametric model and the map is 0.65, which is significantly better than the second-highest CC value of 0.55 and the mean CC value of 0.35 (Fig. 4B). The densities at the 2-fold positions are left uninterpreted (Fig. S7A). Based on the fitting results, hexametric and pentametric capsomeres of the faustovirus inner capsid shell were built. The capsomeres have six or five tilted triangular plate-like Fp150_△NC_ molecules arranged into a screwed-propeller shape (Fig. 4B). The edge β strand group and the central helix group of each Fp150_△NC_ monomer constitute the interior core of the capsomeres, with the edge β strand group located around the quasisymmetry axes. The insertion domains attached to the edge β strand group protrude from the outer surface of the capsomeres and form ringlike structures on the top of the capsomeres. The insertion domains are tightly packed in the top ringlike structure. The edge β strand groups are near the center of the capsomere, while the edge helix group constitutes the edges of the capsomeres and mediates the interactions with neighboring capsomeres through the uninterpreted densities, which are likely constituted by other proteolytic products of the faustovirus pp220-like or pp62-like proteins. However, only a few close contacts (within 5 Å) in between the fitted plate-like domains were detected, which is consistent with the monomeric state of Fp150_△NC_ in solution (Fig. S6C) and suggests that other parts of Fp150 or other components of the capsid may mediate the assembly of Fp150_△NC_.

**FIG 5:**
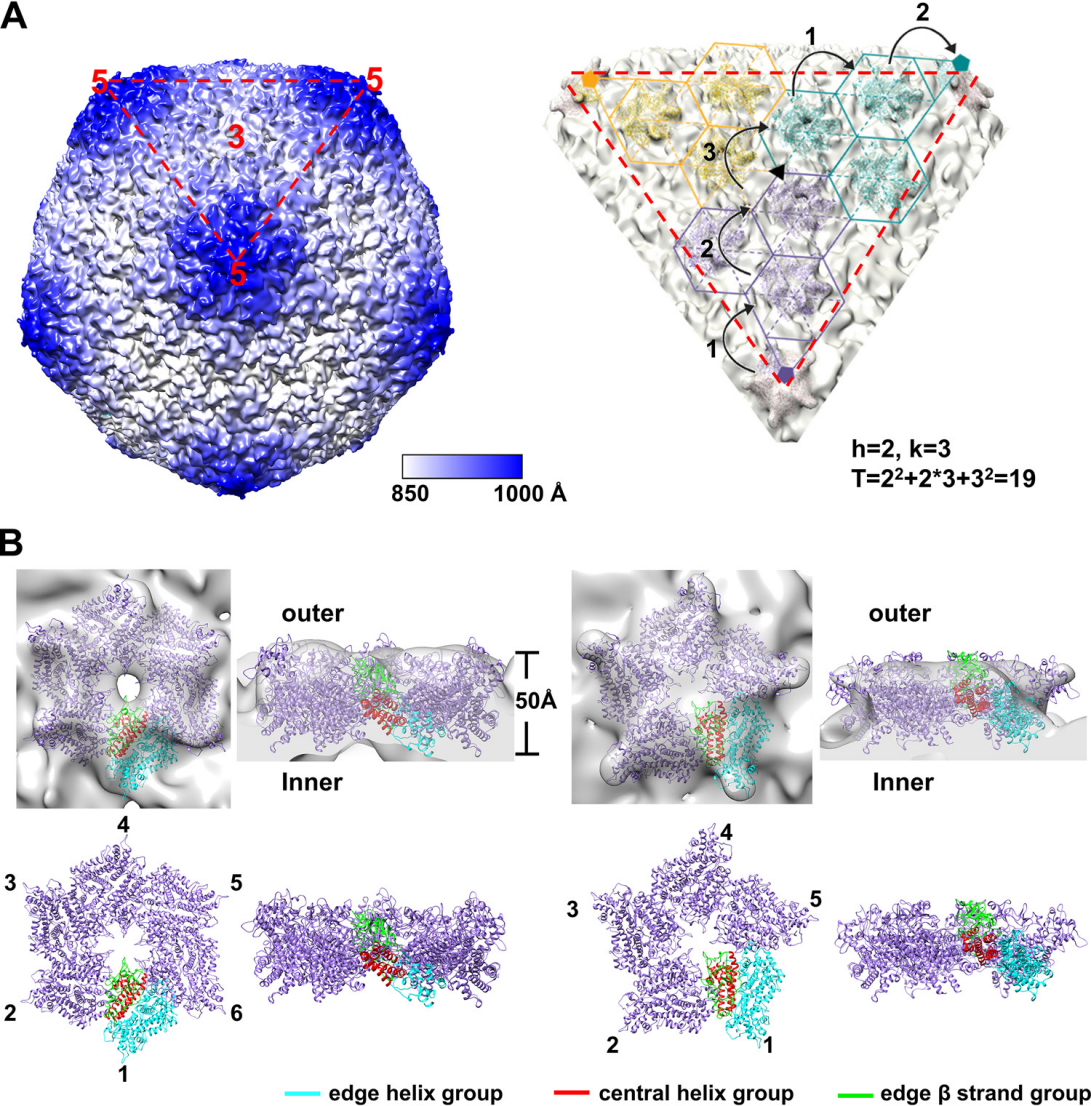
The fitted p150_△NC_ in the cryo-EM map of the ASFV inner core shell. (A) Partial inner core shell structure of ASFV obtained by fitting the hexamer and pentamer of p150_△NC_ into the density map of the ASFV inner core shell extracted from the cryo-EM density map EMD-0815 (4). Voxels of the map are colored according to their radical distances to the particle center. The color scale indicates the color scheme for voxels at different distances to the particle center. One triangular facet is indicated by the red triangle in dashed lines. Positions of the icosahedral symmetr*y* axes are indicated with numbers. The h and k vectors of the hexametric capsomeres are shown on a stripped triangular facet at the right. (B) The hexametric and pentametric capsomers formed by p150_△NC_ are fitted into the density map of the ASFV inner core shell. (B, Right) One hexametric capsomere constituted by six p150_△NC_ molecules is fitted into the density map (EMD-0815) of the ASFV core shell, which was set at a contouring level of 3.9 σ (4). The fitting resulted in a CC value of 0.55, with only the symmetry restrained on each monomer. (B, Left) One pentametric capsomere constituted by six p150_△NC_ molecules is fitted into the density map of the ASFV core shell. The fitting resulted in a CC value of 0.71, with only the symmetry restraint on each monomer. The central helix group, edge helix group, and edge β groups of one monomer are colored red, cyan, and green, respectively. Other monomers are colored purple.

Although the T numbers of the ASFV and faustovirus inner capsids are different (5, 10), given the sequence homology between the polyproteins, the hexametric and pentametric capsomeres for assembling the ASFV and faustovirus inner capsid should be similar. With the Fp150_△NC_ capsomeres as references, we constituted the hexametric and pentametric capsomeres of p150_△NC_, which could be used as the building blocks for the ASFV inner capsid. We then systematically searched the positions of the hexametric and pentametric capsomeres of ASFV p150_△NC_ in the density map of the ASFV inner capsid extracted from the cryo-EM density map of ASFV (EMD-0815) (Fig. 5A). The results showed that the hexamer of ASFV p150_△NC_ could be fitted into quasi-6-fold positions of the map, with a mean CC value of 0.46 and a similar orientation to that of the Fp150_△NC_ capsomere. In contrast, we fitted the hexamer model into the density map at different random positions and in random directions around the quasi-6-fold positions. The mean CC value is only 0.26. Similarly, the pentamers of ASFV p150_△NC_ could be fitted into the 5-fold vertexes of the map, with a CC value of 0.64, while fitting the pentamer model in random orientations at random different positions around the 5-fold vertexes gave a mean CC value of 0.46.

**FIG 6:**
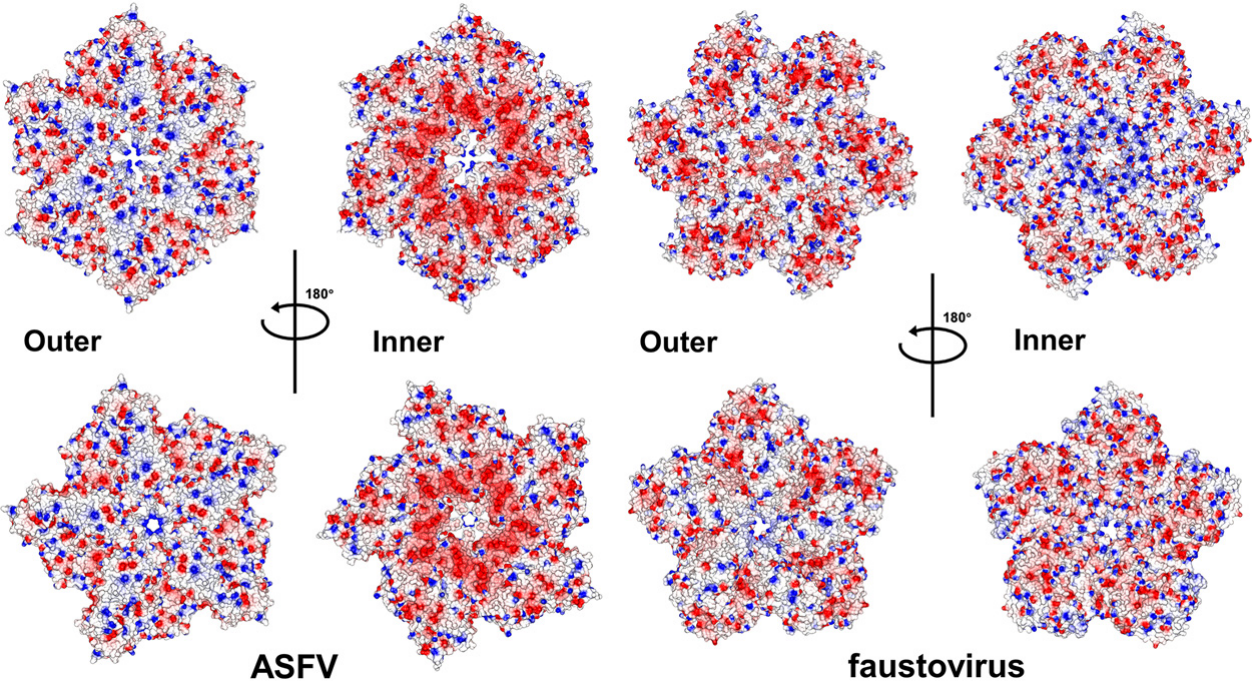
Surface electrostatic potential of the ASFV and faustovirus inner capsid capsomeres. Surface-rendered representations of the ASFV and faustovirus capsomeres. The surfaces are colored according to the electrostatic potential, with red for negative potential and blue for positive potential.

Further fitting of p150_△NC_ monomers in the capsomere by local adjustments with the symmetry restrain improved the CC value to 0.55 for the hexametric capsomere and 0.71 for the pentametric capsomere (Fig. 5B), indicating that the arrangements of Fp150_△NC_ monomers and p150_△NC_ monomers in corresponding capsomeres are slightly different. The surface electrostatic potential analysis of the p150_△NC_ capsomeres showed that the inner surface around the symmetry center is highly negatively charged, which may interact with other potential core shell proteins with a highly positively charged surface (Fig. 6). Of note, surface charges are distinctly different among the capsomeres of the faustovirus and ASFV inner capsids (Fig. 6). Similarly, densities at the 2-fold or quasi-2-fold positions between the hexametric and pentametric capsomeres are left uninterpreted, which may belong to p5, p14, p34, p37, the undetermined rest of p150, or the proteolytic products of pp62 (Fig. S7A).

### Structure of the inner capsid shell.

**FIG 7:**
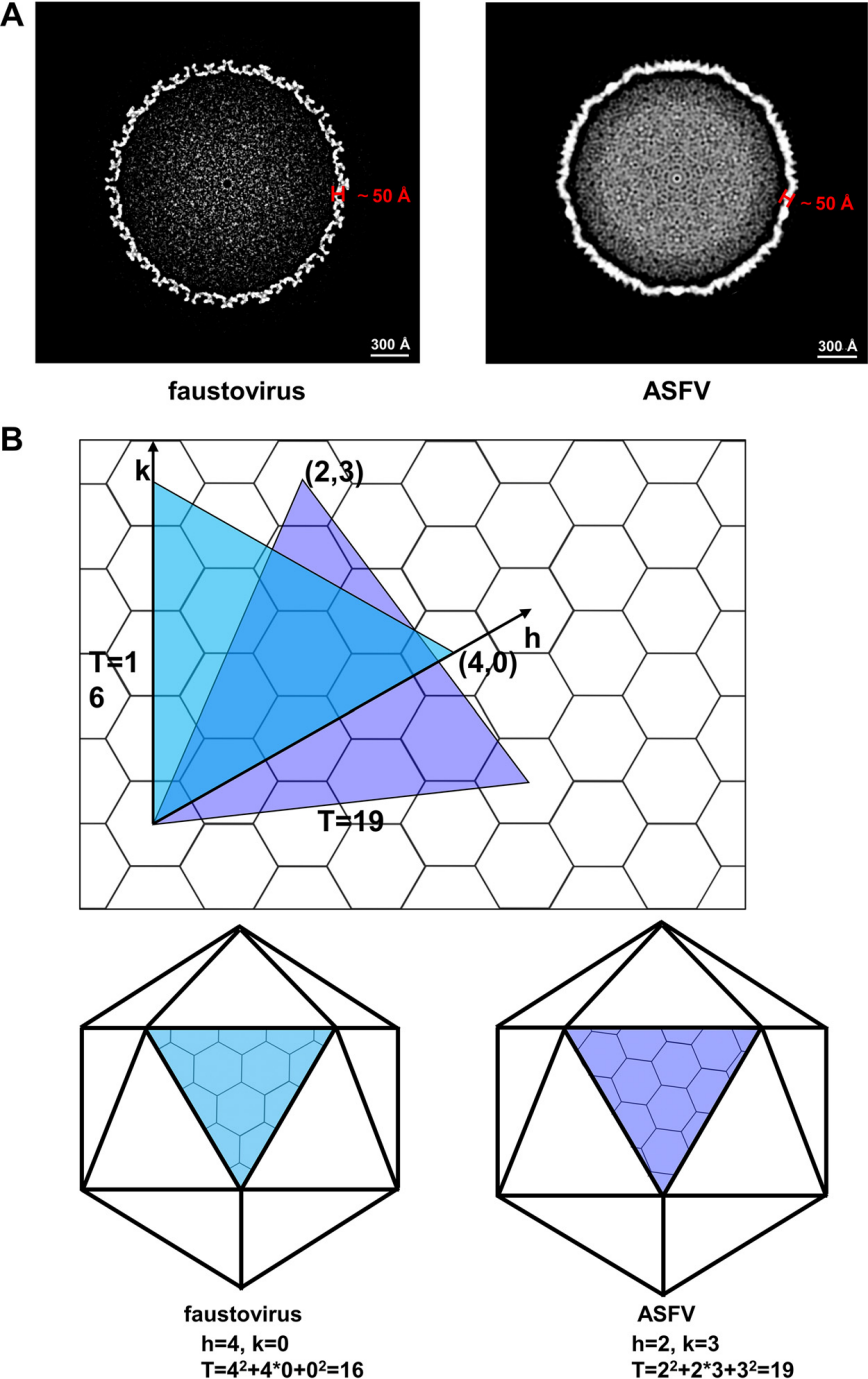
Comparisons of the ASFV and faustovirus inner capsid shells. (A) Images showing the central slices of the icosahedral faustovirus (left) and ASFV (right) inner capsid shells. The thickness of the inner capsid shells is indicated. (B) Comparison of the organization of the capsomeres in the faustovirus and ASFV inner capsid shells.

Based on the fitting results, we could build the partial structures of the ASFV and faustovirus inner capsids (Fig. 4A and Fig. 5A). Unlike the outer capsid that is mainly formed by molecules with a double jelly roll fold and is approximately 90 Å in thickness (6), the inner capsids of ASFV and faustovirus are much thinner and approximately 50 Å in thickness (Fig. 7A), which is comparable to these of the viral capsids assembled by the HK97-like molecules (24). The arrangements of the capsomeres in the inner capsids of ASFV and faustovirus are different and lead to the formation of a T = 19 and a T = 16 icosahedron, respectively (Fig. 7B). The asymmetric unit of the icosahedron has 19 p150_△NC_ molecules and 16 Fp150_△NC_ molecules, which constitute two and a half and three hexametric capsomeres, respectively. The different arrangements may result from subtle different interactions between the capsomeres, which are mediated by the uninterpreted densities.

The uninterpreted densities located between the capsomeres of p150_△NC_ and Fp150_△NC_ are featured with a quasi-2-fold symmetry, around which the uninterpreted densities of the faustovirus inner capsid shell have two protrusions. Similar protrusions could be observed for the inner capsid of ASFV when the contouring level of the map was set to a low value of 2.4 σ (Fig. S7A). Both the N and the C termini of p150_△NC_ and Fp150_△NC_ are in close proximity to the uninterpreted densities (Fig. S7B), suggesting that the terminal domains of p150 and Fp150 might be involved in forming the features around the quasi-2-fold axis. We predicted the N- and C-terminal domain structures of p150 (residues 1 to 267 for p150N and residues 1101 to 1583 for p150C) with AlphaFold. The results showed that p150N contains a single structural domain (Fig. S4), while p150C has two separated domains, including an insertion domain of residues 1260 to 1469 and a base domain of residues 1101 to 1259 and 1479 to 1583 (Fig. S4). Structural predictions of Fp150 N and C domains gave similar results to those of p150 N and C domains (Fig. S4). All the predicted structures have a high percentage of residues with a confidence score of more than 70 (Fig. S4).

To investigate which part of p150 may mediate its oligomerization and interactions with other proteins, we further produced the N- and C-terminal domains (p150N and p150C) of p150 and a truncation mutant, p150_△C_, that contains only residues 1 to 1079 of p150 and does not have the C-terminal domain (Fig. S8 to S9). The recombinant p150N is soluble, and the purified recombinant p150N is a monomer in solution, as shown in the SEC analysis (Fig. S8A). The purified recombinant p150_△C_ is soluble and also exists as a monomer in solution (Fig. S8B). However, Western blotting results showed that only a tiny portion of the recombinant p150C was soluble, and no bands were visible at the corresponding positions on the Coomassie blue-stained SDS-PAGE gel (Fig. S9A and B). We then split the p150C into the base and insertion domains according to the predicted domain structures and produced the recombinant proteins (Fig. S4 and Fig. S9C and D). The p150C insertion domain can be produced in E. coli cells, while expression of the p150C base domain can only be detected in HEK293F cells. The recombinant p150C insertion domain is soluble and is a monomer in solution (Fig. S9C), while differential centrifugations showed that the recombinant p150C base domain is in complex oligomeric states, including SDS-resistant oligomers (Fig. S9D). These combined results suggest that the C-terminal domains of p150 and Fp150 may mediate their oligomerization and interactions with other capsid components.

## DISCUSSION

**FIG 8:**
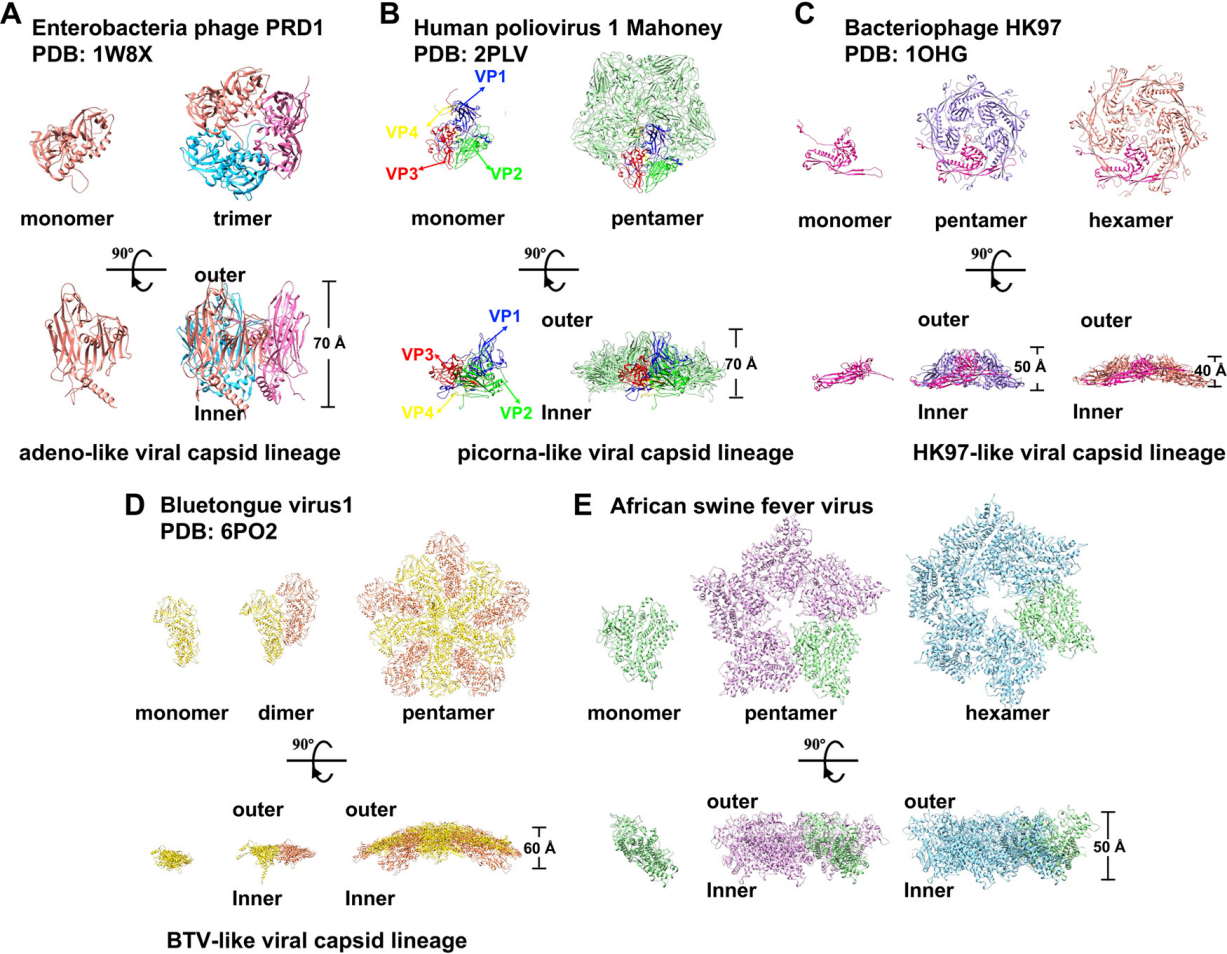
Structural comparisons of capsid proteins from the four viral capsid lineages. Representative structures from the four viral capsid lineages are compared, including the major capsid protein P3 of the bacteriophage PRD1 (A), which belongs to the adeno-like viral capsid lineage; the capsid proteins VP1 to VP4 of the human poliovirus 1 Mahoney (B), which belongs to the picorna-like viral capsid lineage; the major capsid protein gp5 of the bacteriophage HK97 (C), which belongs to the HK97-like viral capsid lineage; the inner core protein VP3 of the bluetongue virus (BTV1) (D), which belongs to the BTV-like viral capsid lineage (24, 43–45); and the ASFV p150 (E).

Although viruses have significant variations in size, genome sequences, and morphology, structures of viral capsid proteins are highly conserved due to their role in making multivalent contacts in a quasiequivalent environment (25). Viral capsid proteins can be grouped into several lineages based on their structure homology (26). So far, four distinct viral capsid lineages have been identified, including the adeno-like, HK97-like, picorna-like, and bluetongue virus (BTV)-like viral capsid lineages (26). The structure of p150 does not belong to any of these capsid structure lineages and thus may represent a new type of capsid assembly subunit (Fig. 8). The SEC results showed that both ASFV p150_△NC_ and faustovirus Fp150_△NC_ exist as a monomer in solution (Fig. S3 and Fig. S6C), rather than forming hexamer or pentamer as in the viral capsid. Further analysis on the recombinant N (residues 1 to 259)- and C (residues 1081 to 1583)-terminal domains, the middle segment (residues 267 to 1101), and p150_△C_ (residues 1 to 1079), which does not have the C-terminal domain, indicated that the multimerization of p150 depends on its C-terminal structure (Fig. S1B; Fig. S8 and S9). The assembly of the inner capsid may also need the aid of other cleavage products of pp220 and pp62, such as p37 and p14.

It is interesting to note that there is still no evidence to show a scaffold-aided assembly of the inner core shell, and structure predictions of the proteolytic cleavage products showed that none of them has a feature consistent with any scaffold protein, which is different from the assembly of ASFV capsid guided by several scaffold-like minor capsid proteins (3, 4). It was shown that during the assembly of the ASFV virion, the core shell seems to be assembled simultaneously with the outer capsid (2, 16). The size of the inner core shell is thus confined by the outer capsid. Initiation and precise control of the inner core shell assembly, which involves interactions with the inner membrane and the genome nucleoid, may have a different mechanism from the assembly of the outer capsid. The association of the polyprotein pp220 with lipid membranes is dependent on its *N*-myristoyl moiety, which may be critical for anchoring the developing core shell onto the viral inner envelope (11). However, there is a remarkable structural difference between ASFV and faustovirus: the latter lacks an inner lipid membrane (10). Assembly of the faustovirus inner capsid involves direct interactions with the outer capsid. In this case, the insertion domain in the ring-shaped top structure of the faustovirus inner shell may be a possible interaction domain with the outer capsid.

As for the proteolytic products of pp62, the crystal structures of p15 and p35 have been reported, and their potential roles in core shell assembly were explored. Among these, p15 forms a three-blade propeller-shaped trimer of three p15 dimers (27, 28). However, the structure of p15 trimer cannot be well fitted in the map of the inner capsid shell or the p150_△NC_-removed residue map of the inner capsid shell, suggesting that p15 is unlikely a component of the inner core shell. P15 is rich in positively charged residues and may be involved in directly binding the genome DNA. P35 is a monomer in solution and was shown to form oligomers under low-pH conditions (29). Structure predictions of other proteolytic products of pp220 and pp62 showed that p34, p14, and p37 are all helix-rich structures. The assembly of these proteins in the inner core shell and the genome nucleoid still needs further studies.

## MATERIALS AND METHODS

### Recombinant protein expression and purification.

The ASFV gene *CP2475L* (strain HLJ/2018) was synthesized from Qinglan (Wuxi, China). *CP2475L* encodes the polyprotein pp220, which, after proteolytic processing, gives rise to proteins p5, p34, p14, p37, and p150 (Fig. 2A). The gene segment encoding protein p150 was cloned into the pCMV vector by using the in-fusion cloning technique with the enzymes Exnase II (Vazyme; catalog no. C112-02). A 10×His-3×Flag tag and a tobacco etch virus (TEV) cleavage site were added to the N terminus of the recombinant p150. The full-length p150 expressed in HEK293F cells was mostly in the insoluble part of the cell lysate. A small portion of the recombinant p150 is soluble and can be purified with anti-Flag resins (GenScript; catalog no. L00432). The protein was eluted with 5 mL Flag peptide and has a concentration of 0.36 mg/mL. The purified p150 shows a molecular weight of ~180 kDa on the SDS-PAGE gels (Fig. S1A in the supplemental material), and most of the proteins were degraded into a stable fragment with a molecular weight of ~80 kDa after storage at 4°C for 1 week (Fig. S1C). The stable fragment of p150 was subjected to mass spectral analysis, and the results showed that all the peptide hits are located in the middle of p150, spanning from residue 226 to residue 1077 (Fig. S2A). According to the mass spectral analysis, five truncation mutants were made and purified by using a similar procedure as for the full-length protein, including mutant 216-1079, mutant 227-1011, mutant 227-1101, mutant 267-1011, and mutant 267-1101, which contain residues 216 to 1079, 227 to 1011, 227 to 1101, 267 to 1011, and 267 to 1101 of p150, respectively (Fig. S2B).

Among the tested truncation mutants, p150_ΔNC_ (mutant 267-1101), which is relatively stable and had a good expression level (Fig. S2B), was selected for large-scale recombinant protein production, purification, and crystallization. In brief, HEK293F cells were cultured in suspension at 37°C with the 293-SIM medium (product M293II; Sino Biological) and 5% CO_2_. For 1-L cell culture, the cells were transfected by a mixture containing 2 mg p150_△NC_ plasmid and 6 mg polyethyleneimine (PEI) at a cell density of 2 × 10^6^ cells/mL. The transfected cells were harvested 48 h posttransfection by centrifugation at 1,000 × *g* for 20 min. The cell pellet was resuspended by a buffer containing 20 mM HEPES at pH 7.4, 300 mM NaCl (resuspension buffer), and cocktail protease inhibitors (Thermo Scientific; catalog no. A32965). The suspended cells were sonicated for 10 min, and the cell lysate was centrifuged for 40 min at 16,000 rpm (JA-25.50 rotor; Beckman). The recombinant p150_ΔNC_ protein in the supernatant was collected and applied to the anti-Flag affinity resin. The resin was washed with the resuspension buffer twice after loading the sample, and then the protein was eluted from the resin with a buffer containing 0.1 mg/mL 3×Flag peptide, 20 mM HEPES at pH 7.4, and 150 mM NaCl.

The eluted p150_△NC_ was mixed with excessive His-tagged TEV protease and was incubated overnight at 4°C. After the TEV digestion, the mixture was applied to cobalt affinity beads (TaKaRa; catalog no. 635653) to remove the cleaved N-terminal 10×His-3×Flag tag, the TEV protease, and p150_△NC_ molecules that still have the tag.

The flowthrough was concentrated and further purified by a Superdex 200 size exclusion column running in a buffer containing 20 mM HEPES at pH 7.4 and 150 mM NaCl (Fig. S3). Peak fractions containing p150_△NC_ were concentrated to ~5 mg/mL for crystallization.

### Sample preparation and mass spectrometry.

Gel bands of proteins were excised for in-gel digestion and identified by mass spectrometry. Briefly, the disulfide bonds of the proteins were reduced with 25 mM dithiothreitol (DTT) and alkylated with 55 mM iodoacetamide. In-gel digestion was performed using the sequencing grade-modified trypsin in 50 mM ammonium bicarbonate at 37°C overnight.

For liquid chromatography-tandem mass spectrometry (LC-MS/MS) analysis, peptides were extracted twice with 1% trifluoroacetic acid in a 50% acetonitrile aqueous solution for 30 min. The peptide extracts were then centrifuged in a SpeedVac to reduce the volume. The peptides were separated by a 120-minute gradient elution at a flow rate of 0.300 μL/min with a Thermo-Dionex Ultimate 3000 high-performance liquid chromatography (HPLC) system, which was directly interfaced with the Thermo Orbitrap Fusion mass spectrometer. The analytical column was a homemade fused silica capillary column (75 μm inner diameter [ID], 150 mm length; Upchurch, Oak Harbor, WA) packed with C_18_ resin (300 A, 5 μm; Varian, Lexington, MA). Mobile phase A consisted of 0.1% formic acid, and mobile phase B consisted of 100% acetonitrile and 0.1% formic acid. The Orbitrap Fusion mass spectrometer was operated in the data-dependent acquisition mode using Xcalibur 3.0 software, and there was a single full-scan mass spectrum in the Orbitrap (350 to 1,550 *m/z*, 120,000 resolution) followed by 3-s data-dependent MS/MS scans in an ion-routing multipole at 30% normalized collision energy (high-energy collisional dissociation [HCD]). The MS/MS spectra from each LC-MS/MS run were searched against the ASFV protein database using the Proteome Discovery searching algorithm (version 1.4).

### Expression and purification of faustovirus p150_ΔNC_.

The faustovirus gene *F-M6_0342* (strain M6) was synthesized from the Qinglan company (Wuxi, China). *F-M6_0342* encodes a polyprotein with a sequence identity of 27.01% to the ASFV polyprotein pp220. To obtain the faustovirus p150_ΔNC_ (Fp150) recombinant protein, four truncation mutants were made and contain residues 251 to 1344, 251 to 1383, 290 to 1400, and 320 to 1383 of Fp150, respectively (Fig. S6A). The encoding gene segment of the truncated protein was cloned into the pCMV vector. A 10×His-3×Flag tag and a TEV cleavage site were added to the N terminus of the recombinant truncation mutants.

Among the tested mutants, mutant 251-1344 (Fp150_△NC_) had the best expression level and stability and was selected for large-scale recombinant protein production and purification (Fig. S6A). HEK293F cells were cultured in suspension at 37°C with the 293-SIM medium and 5% CO_2_. For 1-L cell culture, the cells were transfected by a plasmid mixture containing 2 mg Fp150_△NC_ plasmid and 6 mg PEI at a cell density of 2 × 10^6^ cells/mL. The transfected cells were harvested 48 h posttransfection by centrifugation at 1,000 × *g* for 20 min. The cell pellet was resuspended by a buffer containing 20 mM HEPES at pH 7.4, 300 mM NaCl (resuspension buffer), and cocktail protease inhibitors. The suspended cells were sonicated for 10 min, and the cell lysate was centrifuged for 40 min at 160,000 rpm (JA-25.50 rotor). The recombinant p150_ΔNC_ protein in the supernatant was collected and applied to the anti-Flag affinity resins. The bound protein was washed with the resuspension buffer twice and then eluted from the beads with a buffer containing 0.1 mg/mL 3×flag peptide, 20 mM HEPES at pH 7.4, and 150 mM NaCl.

The elution was mixed with excessive His-tagged TEV protease and digested overnight at 4°C. After the TEV digestion, the mixture was applied to cobalt affinity beads to remove the cleaved N-terminal 10×His-3×Flag tag, the TEV protease, and the uncleaved Fp150_△NC_ protein.

The flowthrough was diluted by 20 mM HEPES at pH 7.4 so that the final concentration of NaCl was 50 mM and then applied directly to a preequilibrated HiTrap Q column (GE Healthcare). The flowthrough that contained Fp150_△NC_ was concentrated to ~0.4 mg/mL for cryo-EM sample preparation (Fig. S6B).

### Crystallization.

All crystals were obtained by hanging-drop vapor diffusion at 18°C using 1 μL protein (5 mg/mL) mixed with an equal volume of well solution. Crystals of the p150_△NC_ protein were grown in 12% (wt/vol) polyethylene glycol 3350 (PEG 3350) and 100 mM *N*-cyclohexyl-2-aminoethanesulfonic acid (CHES) at pH 9.0. The crystals were soaked for 30 s in the well solution containing a final concentration of 30% (vol/vol) glycerol and then were flash frozen in liquid N_2_.

### X-ray data collection, processing, structure determination, refinement, and analysis.

X-ray diffraction data were collected using synchrotron radiation at the Shanghai Radiation Facility beamline BL19U. The data were integrated and scaled with the software XDS (30). The phase problem was solved by molecular replacement using a model generated by AlphaFold (20). The initial phases were then improved by iterative direct method phase extension with Oasis (21), density modifications with Parrot (22), and automatic model building with Buccaneer (23). The program COOT (31) was used for model building and for making adjustments. The program PHENIX (32) was used to refine the structure. The crystals of p150_△NC_ diffracted to ~2.4 Å and belong to space group P1, with two molecules in the asymmetric unit and cell parameters of a = 54.44 Å, b = 65.60 Å, c = 115.01 Å, α = 99.208°, β = 92.705°, and γ = 90.162°. The structure was refined to *R*_work_ and *R*_free_ of 0.2454 and 0.2891.

### Cryo-EM sample preparation, data acquisition, and processing.

We applied 3.5 μL of the purified Fp150_△NC_ at a concentration of ~0.4 mg/mL to glow-discharged holey carbon grids (Quantifoil; Cu; 200 mesh; R1.2/1.3). The grid was blotted for 4.5 s in 100% humidity before being flash-frozen in liquid ethane using a Vitrobot Mark IV (Thermo Fisher). Image data were collected with an FEI Tecnai Spirit electron microscope operating at an acceleration voltage of 120 kV. Images were collected using a Gatan K2 Summit camera at a nominal magnification of ×39,000 with a pixel size of 0.836 Å. Images of Fp150_△NC_ were recorded at a defocus range of −1.3 to −2.0 μm. A total dose of ~50 electrons per Å^2^ was used. A total of 2,660 movie stacks were collected. The movie stacks were aligned using the program MotionCor2 (33) before further processing. The contrast transfer function (CTF) parameters were determined with the program Gctf (34). A total of 1,143,266 Fp150_△NC_ particles were boxed using Gautmatch (https://github.com/JackZhang-Lab) and were subjected to two-dimensional (2D) classification using RELION 3.1 (35). A density map was generated from the predicted atomic structure of Fp150_△NC_ using the molmap function in Chimera (36), which was low-pass filtered to 40 Å and used as the initial model for 3D classifications and refinements with RELION. A total of 83,325 particles were selected for the final 3D refinement without symmetry, which yielded a cryo-EM map at a resolution of approximately 14 Å.

### Recombinant ASFV p150N production and purification.

The N terminal segment (residues 1 to 259) of p150 was cloned into the pET30b vector. A 6×His tag was added to the C terminus of the recombinant p150N. P150N was expressed by IPTG induction in Rosetta cells. The cells were harvested 16 h after isopropyl-β-d-thiogalactopyranoside (IPTG) induction by centrifugation at 4,000 × *g* for 25 min. The cell pellet was resuspended by a buffer containing 20 mM HEPES at pH 7.4, 300 mM NaCl (resuspension buffer), and cocktail protease inhibitors. The suspended cells were sonicated for 20 min, and the cell lysate was centrifuged for 40 min at 16,000 rpm (JA-25.50 rotor). The recombinant p150N protein in the supernatant was collected and applied to the cobalt affinity beads. The bound protein was washed twice with a buffer containing 20 mM HEPES at pH 7.4, 20 mM imidazole, and 300 mM NaCl and then was eluted from the beads with a buffer containing 300 mM imidazole, 20 mM HEPES at pH 7.4, and 150 mM NaCl. The eluted fractions from the cobalt affinity beads were analyzed with SDS-PAGE gels (Fig. S8A). The elution was concentrated and purified by a Superdex 75 size exclusion column in a buffer containing 20 mM HEPES at pH 7.4 and 150 mM NaCl (Fig. S8A).

### Recombinant ASFV p150_△C_ production and purification.

The segment (residues 1 to 1079) of protein p150 was cloned into the vector pCMV. A 3×Flag tag was added to the N terminus of the recombinant p150**_△C_**. P150**_△C_** was expressed and purified using a similar procedure as for p150_△NC_. The eluted fractions from the Flag resins were analyzed with SDS-PAGE gels (Fig. S8B). The eluted fractions were concentrated and then applied to a 15% to 50% (wt/vol) linear glycerol gradient, supplemented with 0.15% glutaraldehyde for gradient fixation (GraFix) (19). The gradient centrifugation was run for 13 h at 240,000 × *g* (Fig. S8B).

### Recombinant protein ASFV p150C production and purification.

The C-terminal segment of protein p150 was cloned into the vector pCMV. A 3×Flag tag was added to the N terminus of the recombinant p150C. Two truncation mutants of p150C were made, including p150C-1, which contains residues 1081 to 1583, and p150C-2, which contains residues 1089 to 1583 of p150 (Fig. S9A). P150C-1 and p150C-2 were expressed and purified using a similar procedure as for p150_△NC_. The eluted fractions from the Flag resins were analyzed with SDS-PAGE gels (Fig. S9B). The precipitate, flowthrough, and elution were also analyzed by Western blotting using an anti-Flag antibody (Sigma; catalog no. F1804) (Fig. S9B).

### Production and purification of ASFV p150C insertion domain.

According to the predicted structure of ASFV p150C by AlphaFold, p150C could be divided into the base and insertion domains (Fig. S4). The insertion domain of ASFV p150C (1260 to 1469) was cloned into the pET30b vector, and a 6×His tag was added to the C terminus of the recombinant p150C insertion domain (Fig. S9A) and expressed by IPTG-induction in Rosetta bacterial cells. The cells were harvested 16 h after IPTG induction by centrifugation at 4,000 × *g* for 20 min. The cell pellet was resuspended by a buffer containing 20 mM HEPES at pH 7.4, 300 mM NaCl (resuspension buffer), and cocktail protease inhibitors. The suspended cells were sonicated for 25 min, and the cell lysate was centrifuged for 40 min at 16,000 rpm (JA-25.50 rotor). The recombinant p150C insertion domain in the supernatant was collected and applied to the cobalt affinity beads. The binding protein was washed with a buffer containing 20 mM HEPES and 20 mM imidazole at pH 7.4 and 300 mM NaCl twice and then was eluted from the beads with a buffer containing 300 mM imidazole, 20 mM HEPES at pH 7.4, and 150 mM NaCl. The elution was concentrated and purified by a Superdex 75 size exclusion column in a buffer containing 20 mM HEPES at pH 7.4 and 150 mM NaCl (Fig. S9C).

### Expression and purification of ASFV p150C base domain.

Different lengths of GS linker (9GS, SGGSGGSGG; 10GS, SGGSGGSGSG; 12GS, SGGSGGSGGSGG) were used to replace the insertion domain of ASFV p150C to obtain the base domain (Fig. S9A). The base domains with different GS linkers were cloned into the vector pCMV. A 3×Flag tag was added to the N terminus of the recombinant p150C base domain. The p150C base domain was expressed by following a similar procedure as for p150_△NC_. The transfected cells were harvested 48 h posttransfection by centrifugation at 1,000 × *g* for 20 min. The cell pellet was resuspended by a buffer containing 20 mM HEPES at pH 7.4, 300 mM NaCl (resuspension buffer), and cocktail protease inhibitors. The suspended cells were sonicated for 10 min, and the cell lysate was centrifuged for 40 min at different rotation speeds (4,000, 7,000, and 16,500 rpm) (JA-25.50 rotor). The supernatant and precipitate at different rotate speeds were collected, and the supernatant centrifuged at 16,500 rpm was applied to the anti-Flag affinity beads. The bound protein was washed with the resuspension buffer twice and then eluted from the beads with a buffer containing 0.1 mg/mL 3×Flag peptide, 20 mM HEPES at pH 7.4, and 150 mM NaCl. The eluted fractions from the Flag beads were analyzed by SDS-PAGE. The supernatant and precipitate collected were also analyzed by Western blotting using an anti-Flag antibody (Fig. S9D).

### Model fitting into the cryo-EM maps.

The model of the Fp150_ΔNC_ monomer predicted by AlphaFold was manually put into the density map (EMD-8145) of the faustovirus inner capsid in Chimera (10, 36). Then, the tool Volume Data-Fit in map in Chimera was used to fit the model into the density map (36). The above-described operation was repeated until all monomers have been fitted into the density map of a hexametric capsomere and the Fp150_ΔNC_ hexamer was made. For the fitting of pentametric capsomere, only one monomer was fitted, and other monomers were generated by using the commands measure symmetry and symmetric fitting in Chimera (10, 36). Then, phenix.real_space_refine was used to improve the fitting by allowing local rigid body adjustments (37, 38). The insertion and the triangular plate-like domains of each Fp150_ΔNC_ monomer were treated as individual rigid bodies. Correlation coefficients (CCs) between the fitted model and the corresponding density map were calculated with phenix.real_space_refine, through which the models were converted into maps at the same resolution as that of the cryo-EM map, and the map CCs were calculated (39). The fitting was improved by maximizing the CC but minimizing the clashes. For the hexametric capsomere, the CCs before and after the real space refinements are 0.60 and 0.65, respectively. To verify that the fitting of the faustovirus p150_△NC_ hexamer, we used e2proc3d.py (40) to move the hexamer to random different positions around the refined fitting position by introducing random translation and rotation to the model. CC was calculated at each position. The second-highest CC value was 0.55, and the mean CC value was 0.35. Fitting refinements of the pentametric capsomere were performed in the same way but with a 5-fold symmetry imposed.

With the Fp150_△NC_ capsomeres as references, we constituted the hexametric and pentametric capsomeres of ASFV p150_△NC_ by using the Tools-Structure Comparison-MatchMarker in Chimera (36). Similarly, the initial fitting of the capsomeres into the density map (EMD-0815) was performed manually in Chimera (4, 36). Then, phenix.real_space_refine was used to improve the fitting by maximizing the CC with the entire hexamer or pentamer treated as a rigid body (38). The fitting of p150_△NC_ was further improved by allowing local rigid body adjustments of the p150_△NC_ monomers with phenix.real_space_refine and with 5-fold symmetry imposed for the pentametric capsomere (36–38). The local adjustments increased the CC value to 0.55 for the hexametric capsomere and to 0.71 for the pentametric capsomere. The fitting positions were also verified by introducing random translation and rotation into the hexamer or pentamer with e2proc3d.py (40), which resulted in an average CC of 0.32 for the hexametric capsomere and 0.50 for the pentametric capsomere.

### Structure prediction.

The structures of ASFV and faustovirus inner core proteins were all predicted by using the AlphaFoldColab (https://colab.research.google.com/github/deepmind/alphafold/blob/main/notebooks/AlphaFold.ipynb) (20).

### Data and material availability.

The atomic coordinate has been deposited into the Protein Data Bank (http://www.rcsb.org) with the accession number 8IGL. The EM map has been deposited into the EM Data Bank with the accession number EMD-35806.
