# Supplementary material for: Structural Insights into the Assembly of the African Swine Fever Virus Inner Capsid
Source: J Virol. 2023 May 16;97(6):e00268-23. doi: 10.1128/jvi.00268-23 (PMC10308890; doi:10.1128/jvi.00268-23)
Supplement: Supplemental file 1 — Table S1 and Fig. S1 to S9. Download jvi.00268-23-s0001.pdf, PDF file, 5.7 MB [file jvi.00268-23-s0001.pdf]

Supplemental Material

Table S1 Data collection, refinement, and model statistics

Resolution range	32.54 - 2.4 (2.486 - 2.4)
Space group	P 1
Unit cell	54.44 65.6 115.01 99.208 92.705 90.162
Total reflections	103496 (10705)
Unique reflections	58403 (5913)
Redundancy	1.8 (1.8)
Completeness (%)	95.11 (96.41)
Mean I/sigma(I)	11.60 (4.07)
R-meas	0.07371 (0.2459)
CC1/2	0.996 (0.953)
CC*	0.999 (0.988)
Reflections used in refinement	58343 (5913)
Reflections used for R-free	2937 (312)
R-work	0.2454 (0.2647)
R-free	0.2891 (0.3076)
R.m.s deviations:	
Bonds (Å)	0.005
Angles (º)	0.83
Wilson B-factor (Å^2)	9.69
Average B-factor (Å^2)	16.32
Protein	16.39
Solvent	15.21
Number of atoms	
Protein	25447
Non-protein	725
Ramachandran analysis:	
Favored (%)	95.86%
Allowed (%)	4.14%
Outliers (%)	0.00%

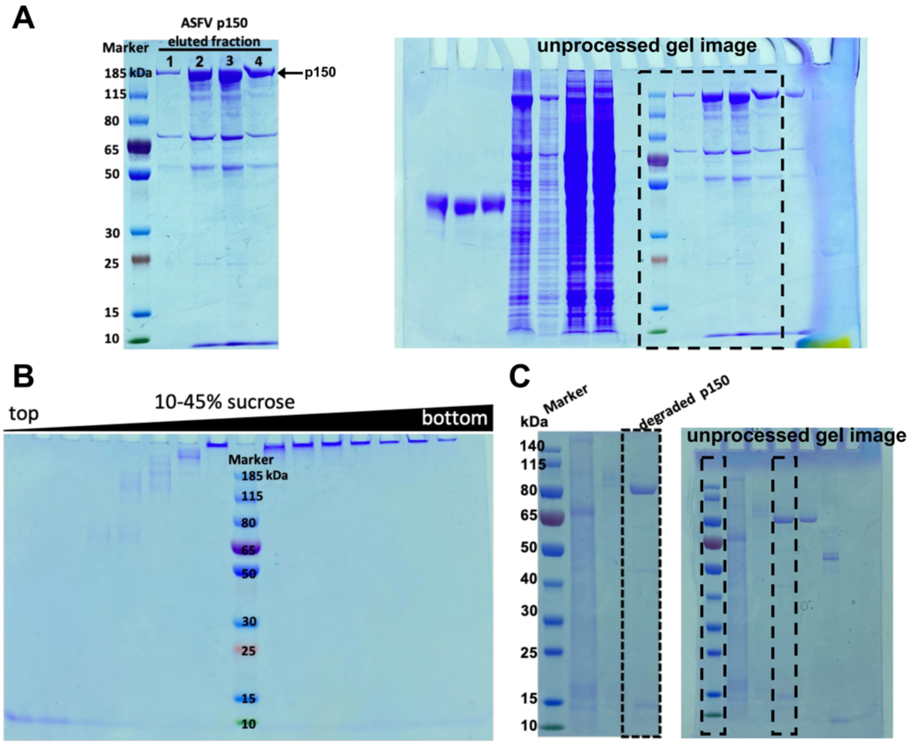
A
ASFV p150
eluted fraction
Marker
1
2
3
4
185 kDa
115
80
65
50
30
25
15
10
p150
unprocessed gel image
B
top
10-45% sucrose
bottom
Marker
185 kDa
115
80
65
50
30
25
15
10
C
Marker
degraded p150
kDa
140
115
80
65
50
40
30
25
15
10
unprocessed gel image

Figure S1 Characterization of the recombinant full-length p150 and p150$_{\Delta NC}$.

A. SDS-PAGE gel analysis of the soluble recombinant full-length ASFV gp150, which has a molecular weight of approximately 185 kDa.

B. GraFix analysis of the soluble recombinant full-length ASFV gp150 with a 10-45% sucrose gradient from top to bottom. Most of the proteins are in a highly oligomerized state.

C. SDS-PAGE gel analysis showing the stable fragment of p150 after degradation.

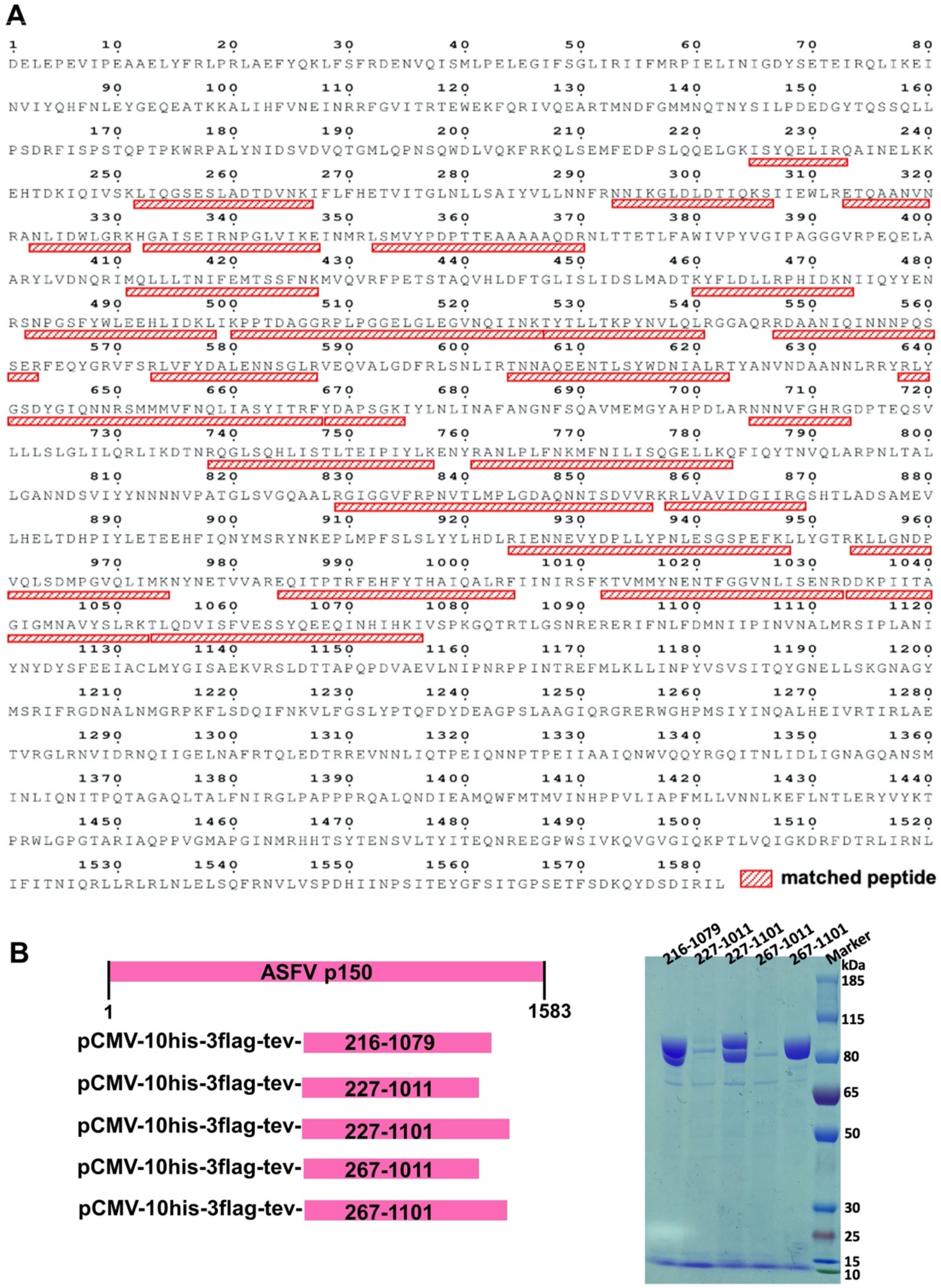
A
DELEPEVIPEAAELYFRLPRLAEFYQKLFSFRDENVQISMLPELEGIFSGLIRIIFMRPIELINIGDYSETEIRQLIKEI
NVIYQHFNLEYGEQEATKKALIHFVNEINRRFGVITRTEWEKFQRIVQEARTMNDFGMMNQTNYSILPDEDGYTQSSQLL
PSDRFISPSTQPTPKWRPALYNIDSVDVQTGMLQPNSQWDLVQKFRKQLSEMFEDPSLQQELGKISYQELIRQAINELKK
EHTDKIQIVSKLIQGSESLADTDVNKIFLFHETVITGLNLLSAIYVLLNNFRNNIKGLDLDTIQKSIIEWLRETQAANVN
RANLIDWLGRKHGAISEIRNPGLVIKEINMRLSMVYPDPTTEAAAAAQDRNLTTETLFAWIVPYVGIPAGGGVRPEQELA
ARYLVDNQRIMQLLLTNIFEMTSSFNKMVQVRFPETSTAQVHLDFTGLISLIDSLMADTKYFLDLLRPHIDKNIIQYYEN
RSNPGSFYWLEEHLIDKLIKPPTDAGGRPLPGGELGLEGVNQIINKTYTLLTKPYNVLQLRGGAQRRDAANIQINNNPQS
SERFEQYGRVFSRLVFYDALENNSGLRVEQVALGDFRLSNLIRTNNAQEENTLSYWDNIALRTYANVNDAANNLRRYRLY
GSDYGIQNNRSMMMVFNQLIASYITRFYDAPSGKIYLNLINAFANGNFSQAVMEMGYAHPDLARNNNVFGHRGDPTEQSV
LLLSLGLILQRLIKDTNRQGLSQHLISTLTEIPIYLKENYRANLPLFNKMFNILISQGELLKQFIQYTNVQLARPNLTAL
LGANNDSVIYYNNNNVPATGLSVGQAALRGIGGVFRPNVTLMPLGDAQNNTSDVVRKRLVAVIDGIIRGSHTLADSAMEV
LHELTDHPIYLETEEHFIQNYMSRYNKEPLMPFSLSLYYLHDLRIENNEVYDPLLYPNLESGSPEFKLLYGTRKLLGNDP
VQLSDMPGVQLIMKNYNETVVAREQITPTRFEHFYTHAIQALRFIINIRSFKTVMMYNENTFGGVNLISENRDDKPIITA
GIGMNAVYSLRKTLQDVISFVESSYQEEQINHIHKIVSPKGQTRTLGSNRERERIFNLFDMNIIPINVNALMRSIPLANI
YNYDYSFEEIACLMYGISAEKVRSLDTTAPQPDVAEVLNIPNRPPINTREFMLKLLINPYVSVSITQYGNELLSKGNAGY
MSRIFRGDNALNMGRPKFLSDQIFNKVLFGSLYPTQFDYDEAGPSLAAGIQRGRERWGHPMSIYINQALHEIVRTIRLAE
TVRGLRNVIDRNQIIGELNAFRTQLEDTRREVNNLIQTPEIQNNPTPEIIAAIQNWVQQYRGQITNLIDLIGNAGQANSM
INLIQNITPQTAGAQLTALFNIRGLPAPPPRQALQNDIEAMQWFMTMVINHPPVLIAPFMLLVNNLKEFLNTLERYVYKT
PRWLGPGTARIAQPPVGMAPGINMRHHTSYTENSVLTYITEQNREEGPWSIVKQVGVGIQKPTLVQIGKDRFDTRLIRNL
IFITNIQRLLRLRLNLELSQFRNVLVSPDHIINPSITEYGFSITGPSETFSDKQYDSDIRIL
matched peptide
B
ASFV p150
1
1583
pCMV-10his-3flag-tev-
216-1079
pCMV-10his-3flag-tev-
227-1011
pCMV-10his-3flag-tev-
227-1101
pCMV-10his-3flag-tev-
267-1011
pCMV-10his-3flag-tev-
267-1101
216-1079
227-1011
227-1101
267-1011
267-1101
Marker
kDa
185
115
80
65
50
30
25
15
10

Figure S2 Identification of the p150 fragment from degradation and construction of p150 truncations

A. Sequence of ASFV p150 is shown. The white bars with red stripes below the sequence indicate peptide fragments identified in the stable fragment of p150 by mass spectrum.

B. Left: schematic diagrams showing the different constructs of p150. Right: an SDS-PAGE gel showing the expression of p150 truncation mutants.

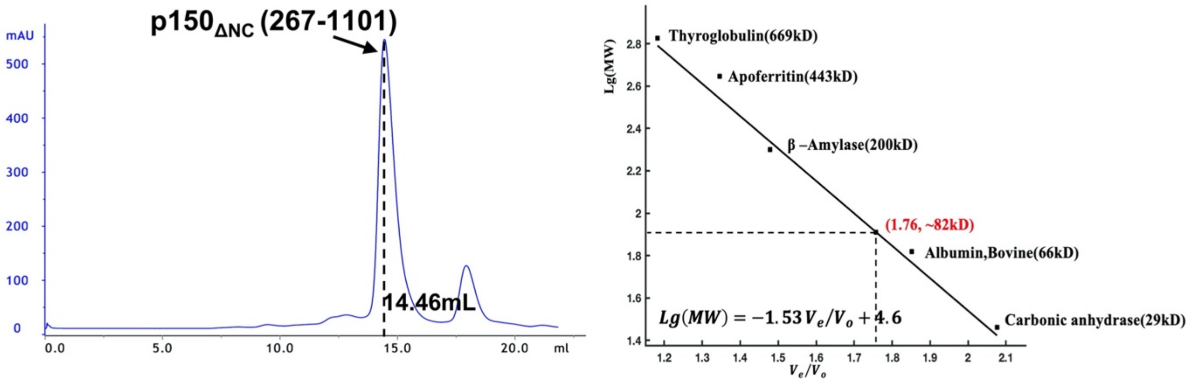

p150ΔNC (267-1101)
mAU
500
400
300
200
100
0
14.46mL
0.0
5.0
10.0
15.0
20.0
ml
Lg(MW)
Thyroglobulin(669kD)
Apoferritin(443kD)
β –Amylase(200kD)
(1.76, ~82kD)
Albumin,Bovine(66kD)
Carbonic anhydrase(29kD)
Lg(MW) = −1.53 Ve/Vo + 4.6
Ve/Vo

Figure S3 Size exclusion chromatographic (SEC) analysis of p150$_{\triangle NC}$. Left: A SEC elution profile of p150$_{\triangle NC}$; right: A plot of lg (MW) as a function of Ve/Vo. Vo is the void volume of the column. Ve is the elution volume for each sample, including p150$_{\triangle NC}$ and the standard protein samples. The estimated molecular weight for p150$_{\triangle NC}$ in solution is ～82 kDa, which is consistent with the molecular weight of a p150$_{\triangle NC}$ monomer.

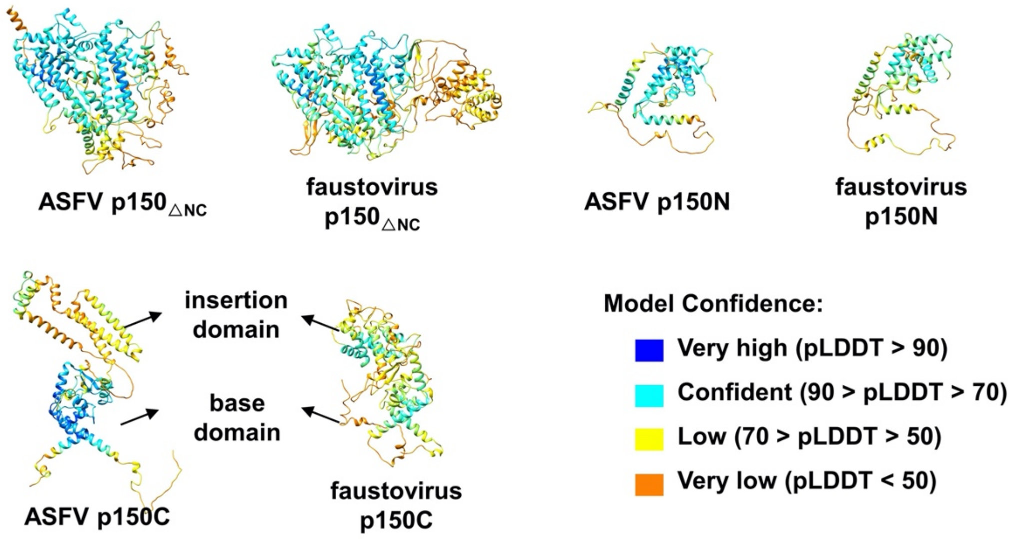

ASFV p150△NC
faustovirus p150△NC
ASFV p150N
faustovirus p150N
insertion domain
base domain
ASFV p150C
faustovirus p150C
Model Confidence:
Very high (pLDDT > 90)
Confident (90 > pLDDT > 70)
Low (70 > pLDDT > 50)
Very low (pLDDT < 50)

Figure S4 AlphaFold structure predictions of the ASFV and faustovirus p150.

The polypeptide chains are colored according to the confidence scores of the residues (20).

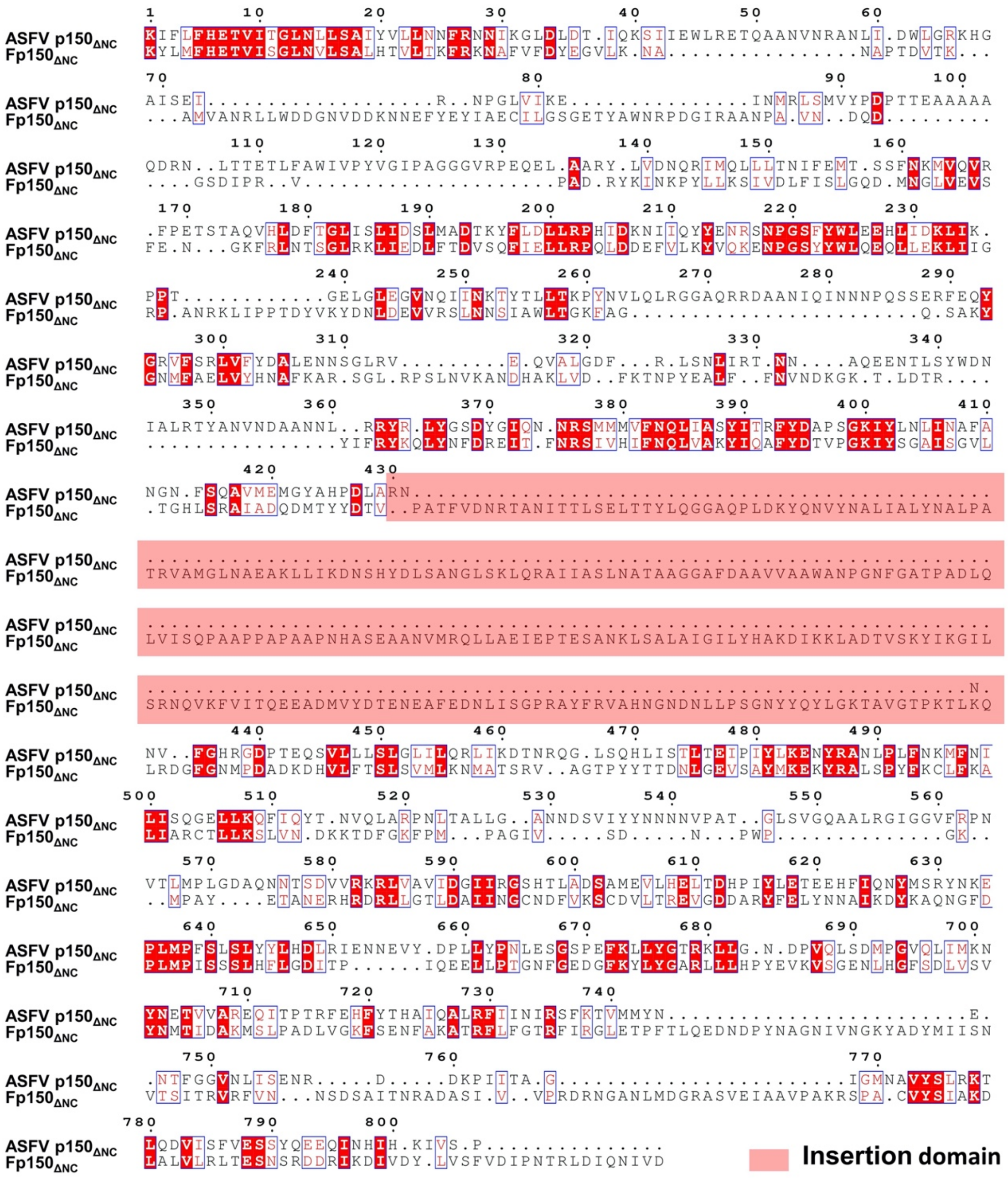
ASFV p150ΔNC
Fp150ΔNC
Insertion domain

Figure S5 Sequence alignments of the ASFV p150$_{\Delta NC}$ and the faustovirus p150$_{\Delta NC}$ (Fp150$_{\Delta NC}$). The sequence in red background belongs to the insertion domain of Fp150$_{\Delta NC}$.

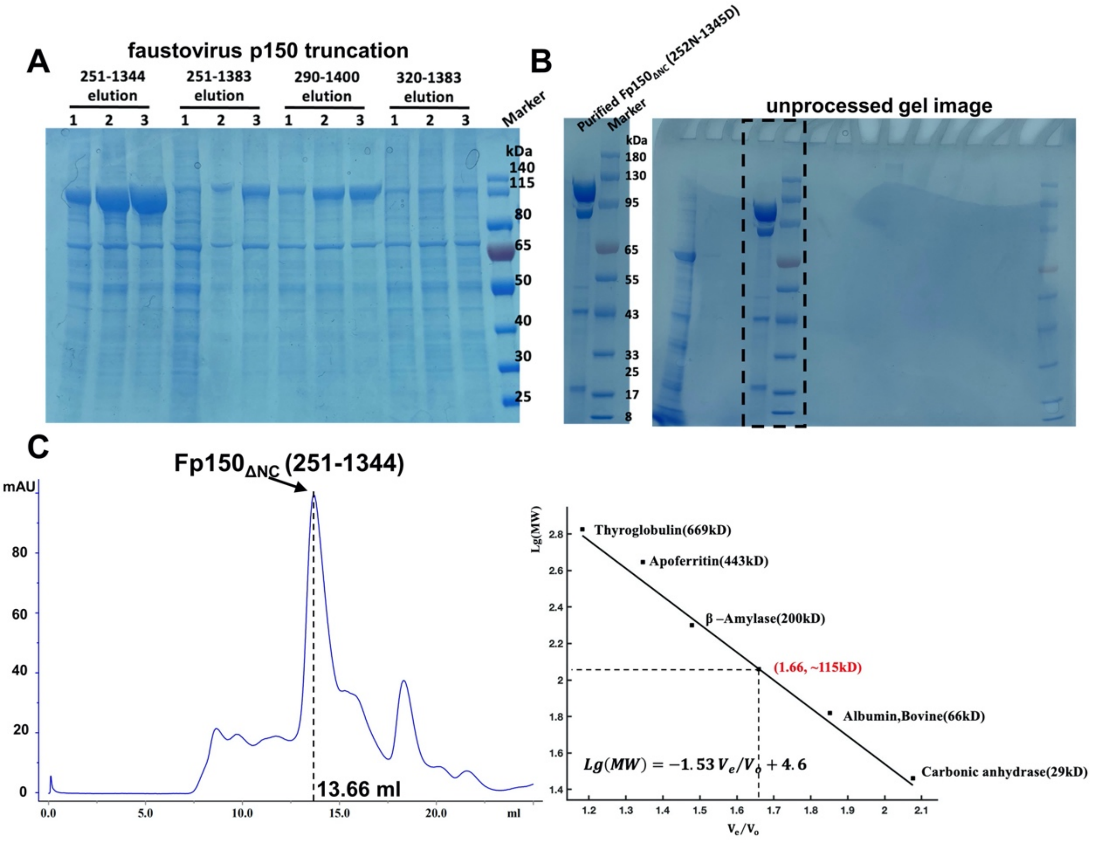
A
faustovirus p150 truncation
251-1344 elution
251-1383 elution
290-1400 elution
320-1383 elution
1 2 3
Marker
kDa
140
115
80
65
50
40
30
25
B
Purified Fp150ΔNC (252N-1345D)
Marker
unprocessed gel image
kDa
180
130
95
65
55
43
33
25
17
8
C
Fp150ΔNC (251-1344)
mAU
80
60
40
20
0
13.66 ml
0.0
5.0
10.0
15.0
20.0
ml
Lg(MW)
Thyroglobulin(669kD)
Apoferritin(443kD)
β −Amylase(200kD)
(1.66, ~115kD)
Albumin,Bovine(66kD)
Carbonic anhydrase(29kD)
Lg(MW) = −1.53 Ve/Vo + 4.6
Ve/Vo

Figure S6 Expression and purification of the faustovirus p150 truncation mutants.

A. SDS-PAGE gel analysis of the expression of the faustovirus p150 truncation mutants.

B. SDS-PAGE of purified faustovirus $p150_{\Delta NC}$ (251-1344).

C. Purification of faustovirus $p150_{\Delta NC}$ (251-1344) with SEC. Left: A SEC elution profile of $Fp150_{\Delta NC}$; right: A plot of lg (MW) as a function of Ve/Vo. Vo is the void volume of the column. Ve is the elution volume for each sample, including $Fp150_{\Delta NC}$ and the standard protein samples. The estimated molecular weight for $Fp150_{\Delta NC}$ in solution is ~ 115 kDa, which is consistent with the molecular weight of a $Fp150_{\Delta NC}$ monomer.

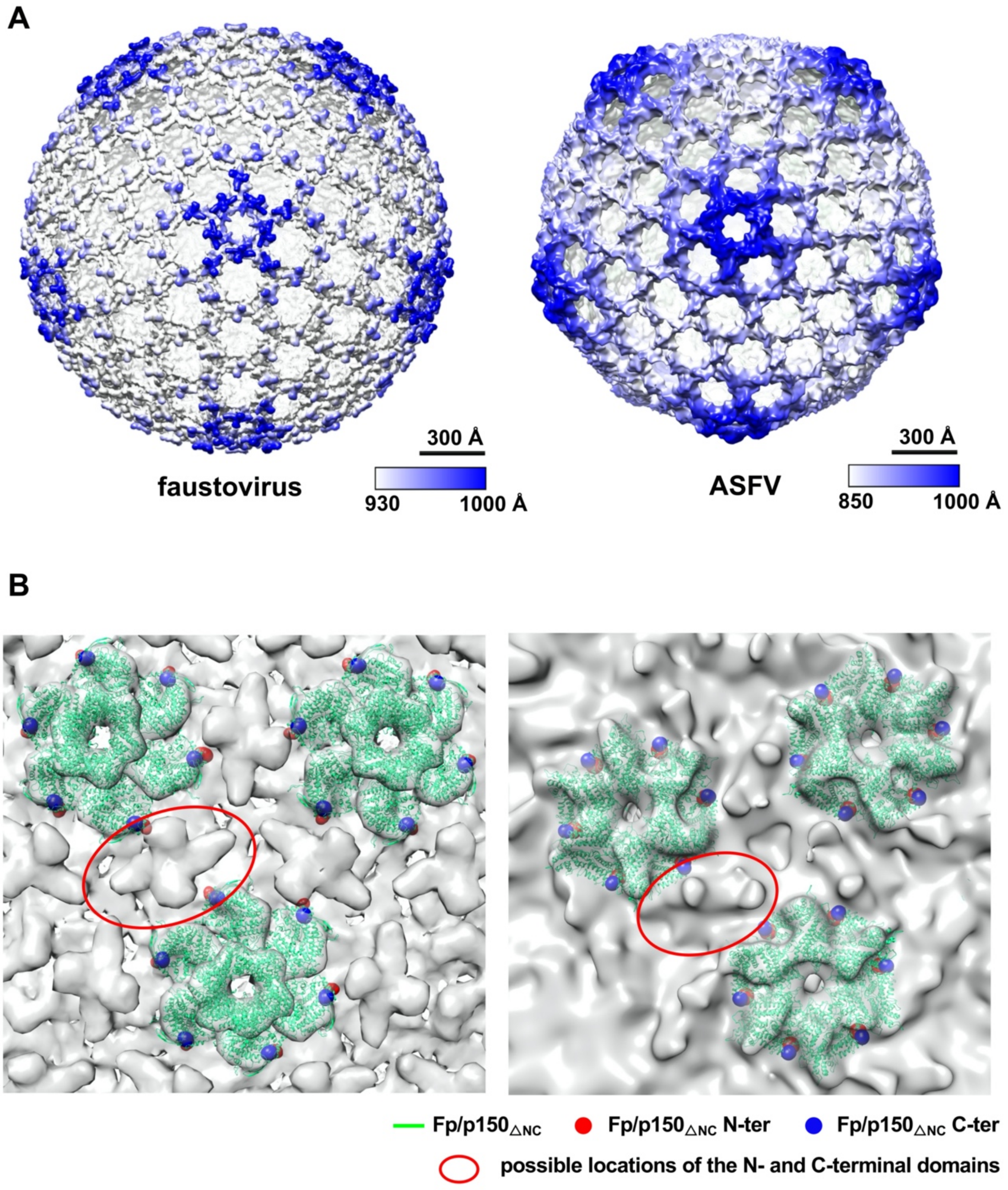

A
300 Å
faustovirus
930
1000 Å
300 Å
ASFV
850
1000 Å
B
Fp/p150△NC
Fp/p150△NC N-ter
Fp/p150△NC C-ter
possible locations of the N- and C-terminal domains

Figure S7 The uninterpreted densities of the faustovirus (Left) and ASFV (Right) inner capsids.

A. The residue density maps of the faustovirus (EMD-8145) and ASFV (EMD-0815) inner capsids were contouring at 2.4 σ and 3.4 σ, respectively (4,10). The map is colored according to the radius from the particle center. The color scale indicates different colors for voxels at different distances to the particle center.

B. Possible locations of the N- and C-terminal domains of Fp/p150. The Fp/p150$_{\Delta NC}$ hexametric capsomers are colored light green. The N and C termini of each Fp/p150$_{\Delta NC}$ monomer are labeled with a red and a blue ball, respectively. The red circles indicate the possible locations of the N- and C-terminal domains.

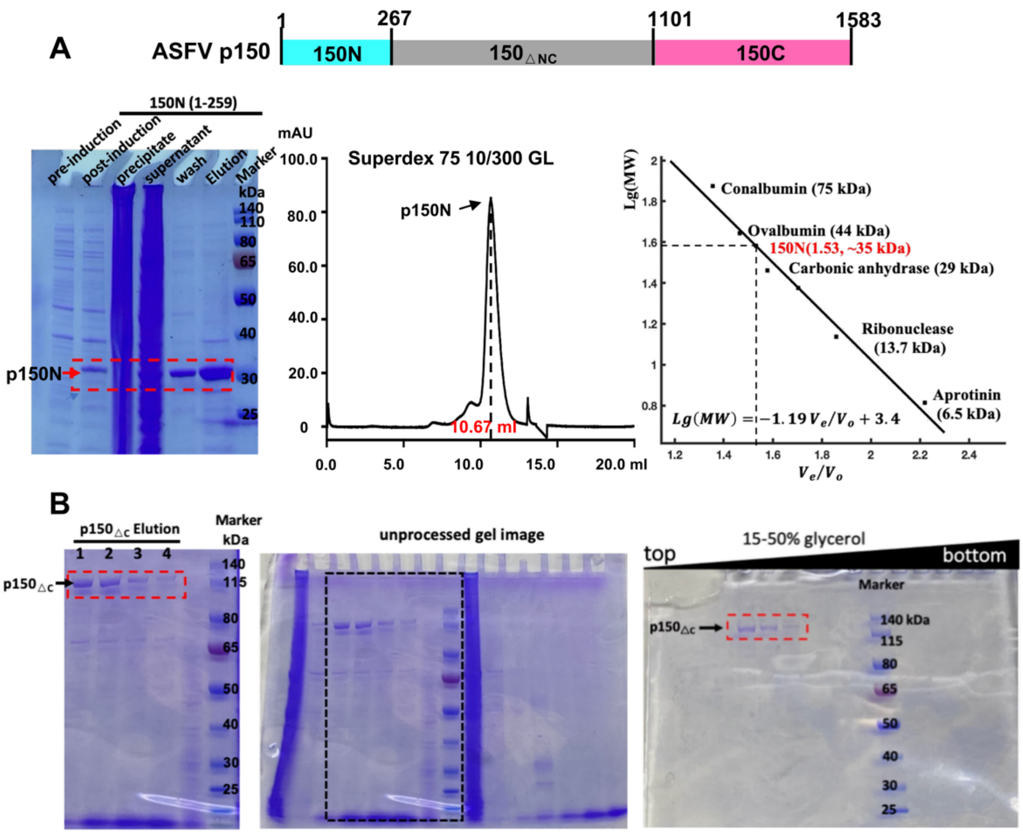
A
ASFV p150
1
267
1101
1583
150N
150△NC
150C
150N (1-259)
pre-induction
post-induction
precipitate
supernatant
wash
Elution
Marker
kDa
140
110
80
65
50
40
30
25
p150N
mAU
Superdex 75 10/300 GL
100.0
80.0
60.0
40.0
20.0
0
p150N
10.67 ml
0.0
5.0
10.0
15.0
20.0 ml
Lg(MW)
Conalbumin (75 kDa)
Ovalbumin (44 kDa)
150N(1.53, ~35 kDa)
Carbonic anhydrase (29 kDa)
Ribonuclease
(13.7 kDa)
Aprotinin
(6.5 kDa)
Lg(MW) = −1.19 Ve/Vo + 3.4
Ve/Vo
B
p150△c Elution
1 2 3 4
Marker
kDa
140
115
80
65
50
40
30
25
p150△c
unprocessed gel image
top
15-50% glycerol
bottom
Marker
140 kDa
115
80
65
50
40
30
25
p150△c

Figure S8 Characterization of the recombinant ASFV p150N and p150$_{\triangle C}$.

A. Top: a schematic diagram showing the potential domain arrangement of ASFV p150. Bottom: SDS-PAGE gel analysis of p150N expressed in *E. coil* and a SEC elution profile of p150N. A plot of lg (MW) as a function of Ve/Vo. Vo is the void volume of the column. Ve is the elution volume for each sample, including p150N, p150C-insertion domain, and the standard protein samples. The estimated molecular weight for p150N in solution is approximately 35 kDa, which is consistent with the molecular weight of a p150N monomer.

B. Left: SDS-PAGE gel analysis of the expression of the ASFV p150$_{\triangle C}$ truncation mutants. Right: GraFix analysis of the soluble recombinant ASFV p150$_{\triangle C}$ with a 15-50% glycerol gradient. Fractions from the top to the bottom of the gradient were analyzed with the SDS PAGE gel. The proteins are located in the top fractions and in a monomer state.

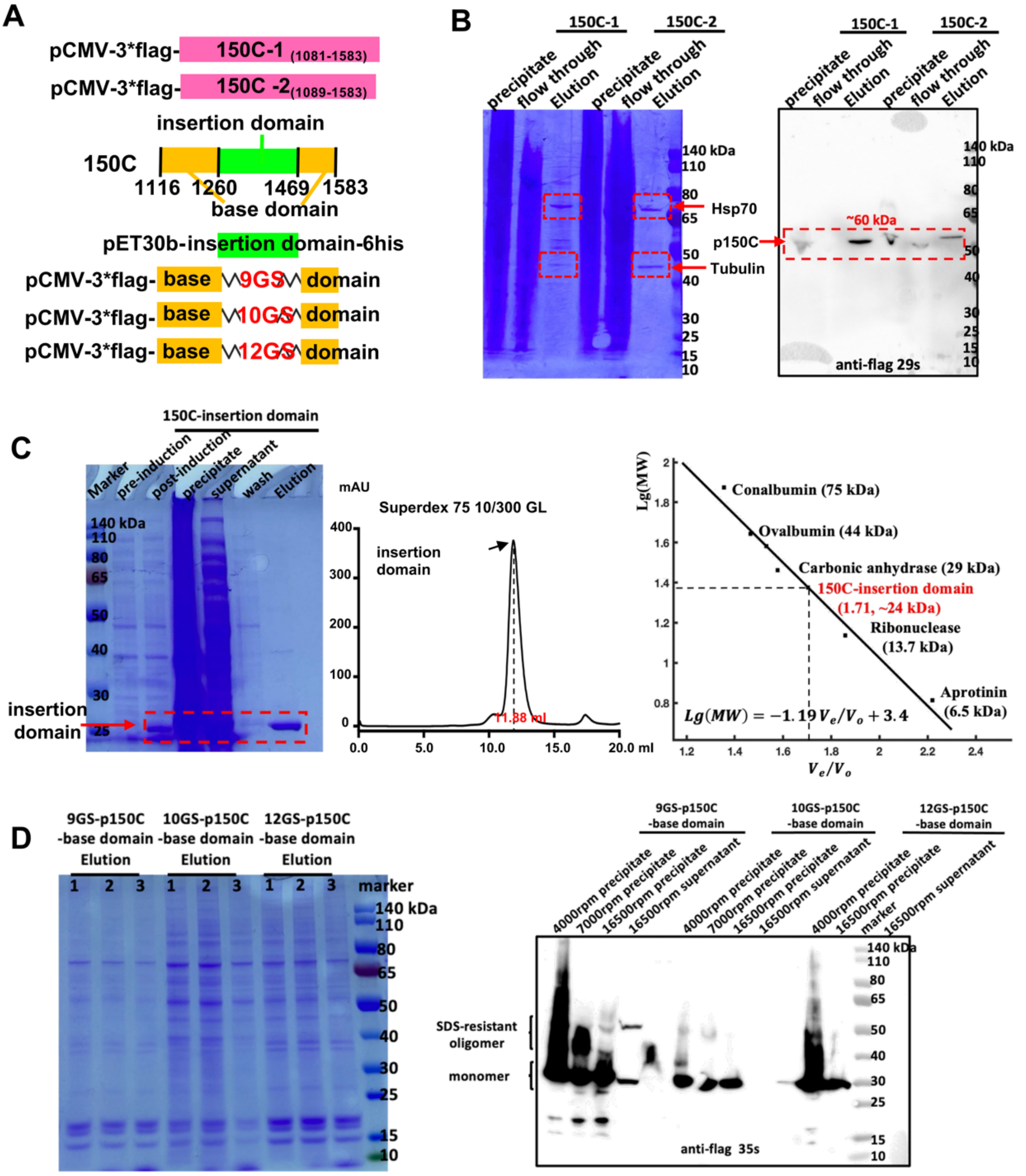

A
pCMV-3*flag- 150C-1 (1081-1583)
pCMV-3*flag- 150C -2(1089-1583)
insertion domain
150C
1116 1260 1469 1583
base domain
pET30b-insertion domain-6his
pCMV-3*flag- base 9GS domain
pCMV-3*flag- base 10GS domain
pCMV-3*flag- base 12GS domain
B
150C-1
150C-2
precipitate
flow through
Elution
Hsp70
Tubulin
p150C
~60 kDa
anti-flag 29s
C
150C-insertion domain
Marker
pre-induction
post-induction
precipitate
supernatant
wash
Elution
insertion domain
mAU
Superdex 75 10/300 GL
11.88 ml
Conalbumin (75 kDa)
Ovalbumin (44 kDa)
Carbonic anhydrase (29 kDa)
150C-insertion domain
(1.71, ~24 kDa)
Ribonuclease
(13.7 kDa)
Aprotinin
(6.5 kDa)
Lg(MW)
Lg(MW) = −1.19 Ve/Vo + 3.4
Ve/Vo
D
9GS-p150C -base domain Elution
10GS-p150C -base domain Elution
12GS-p150C -base domain Elution
marker
9GS-p150C -base domain
10GS-p150C -base domain
12GS-p150C -base domain
4000rpm precipitate
7000rpm precipitate
16500rpm precipitate
16500rpm supernatant
marker
SDS-resistant oligomer
monomer
anti-flag 35s

Figure S9 Characterization of the recombinant ASFV p150C.

A: Schematic diagrams showing different truncation mutants of ASFV p150C and the base and insertion domains of ASFV p150C.

B: SDS-PAGE gel and western blotting analysis of the expression of the ASFV p150C truncation mutants.

C: SDS-PAGE gel analysis of the p150C insertion domain expressed in *E. coil* and a SEC elution profile of the p150C insertion domain; The estimated molecular weight of the p150C insertion domain in solution is approximately 24 kDa, which is consistent with the molecular weight of a monomer.

D: SDS-PAGE gel and western blotting analysis of the expression of the ASFV p150C-base domain truncation mutants. Different GS (9GS: SGGSGGSGG, 10GS: SGGSGGSGSG, 12GS: SGGSGGSGGSGG) linkers replaced the insertion domains in different constructs.
